# Generation of Superoxide by OeRbohH, a NADPH Oxidase Activity During Olive (*Olea europaea* L.) Pollen Development and Germination

**DOI:** 10.3389/fpls.2019.01149

**Published:** 2019-09-19

**Authors:** María José Jimenez-Quesada, José Angel Traverso, Martin Potocký, Viktor Žárský, Juan de Dios Alché

**Affiliations:** ^1^Plant Reproductive Biology and Advanced Microscopy Laboratory, Department of Biochemistry, Cellular and Molecular Biology of Plants, Estación Experimental del Zaidín (CSIC), Granada, Spain; ^2^Institute of Experimental Botany of the Czech Academy of Sciences, Prague, Czechia; ^3^Department of Experimental Plant Biology, Faculty of Science, Charles University in Prague, Prague, Czechia

**Keywords:** NADPH oxidase, NOX, pollen, Rboh, sexual plant reproduction, olive

## Abstract

Reactive oxygen species (ROS) are produced in the olive reproductive organs as the result of intense metabolism. ROS production and pattern of distribution depend on the developmental stage, supposedly playing a broad panel of functions, which include defense and signaling between pollen and pistil. Among ROS-producing mechanisms, plasma membrane NADPH-oxidase activity is being highlighted in plant tissues, and two enzymes of this type have been characterized in *Arabidopsis thaliana* pollen (RbohH and RbohJ), playing important roles in pollen physiology. Besides, pollen from different species has shown distinct ROS production mechanism and patterns of distribution. In the olive reproductive tissues, a significant production of superoxide has been described. However, the enzymes responsible for such generation are unknown. Here, we have identified an Rboh-type gene (OeRbohH), mainly expressed in olive pollen. OeRbohH possesses a high degree of identity with RbohH and RbohJ from *Arabidopsis*, sharing most structural features and motifs. Immunohistochemistry experiments allowed us to localize OeRbohH throughout pollen ontogeny as well as during pollen tube elongation. Furthermore, the balanced activity of tip-localized OeRbohH during pollen tube growth has been shown to be important for normal pollen physiology. This was evidenced by the fact that overexpression caused abnormal phenotypes, whereas incubation with specific NADPH oxidase inhibitor or gene knockdown lead to impaired ROS production and subsequent inhibition of pollen germination and pollen tube growth.

## Introduction

Pollen–pistil interaction is recognized as a key aspect of sexual plant reproduction. The pollen grains must undergo a tightly controlled sequence of physiological events after landing on compatible stigmatic papillae. These processes involve the initial pollen rehydration and germination, the pollen tube growth through the female tissues, and the final interaction with the embryo sac to eventually achieve the double fertilization and generate the progeny. To assure the success of the sexual plant reproduction, pollen and the female tissues of the pistil must accomplish an efficient cross talk, which involves a large number of signaling mechanisms. Redox regulation and signaling are now proposed as a crucial mechanism able to manage different aspects of the sexual plant reproduction, where ROS and NO molecules seem to act as mediators in such an interchange of information between pollen and pistil tissues (Traverso et al., 2013b; Domingos et al., 2015).

NADPH oxidase enzymes are eukaryotic proteins able to catalyze the physiological generation of the short-lived superoxide radical (O_2_^•−^) throughout membranes (Lambeth, 2004), which is rapidly dismutated leading to H_2_O_2_ accumulation (Lamb and Dixon, 1997). This protein family shares six transmembrane central domains, two heme-binding sites, and a long cytoplasmic C-terminal end owning FAD- and NADPH-binding domains. In addition, Ca^2+^-binding EF-hand motifs in the N-terminus are a distinctive feature of plant NADPH oxidases as well as NOX5 and DUOX human NADPH oxidases (Bedard et al., 2007). In *Arabidopsis thaliana*, 10 NADPH oxidase homologues are encoded (Torres et al., 1998; Dangl and Jones, 2001), which are also designed as “*respiratory burst oxidase homologs*” (*Rbohs*).

Rbohs play crucial roles in a broad range of responses to biotic interaction (pathogenic or symbiotic) as well as in the response to different kinds of abiotic stresses and adaptation mechanisms. Moreover, Rbohs have been shown to be involved in cell growth (diffuse or polarized) and other developmental events (Marino et al., 2012). Cell-to-cell communication over long distances in plants has also been shown to be mediated by Rboh-derived O_2_^•−^ (Miller et al., 2009). Among these enzymes, *Arabidopsis* RbohH and RbohJ seem to be specifically expressed in pollen and stamens (Sagi and Fluhr, 2006). Moreover, NADPH oxidase-produced ROS are detected through pollen tube growth, subsequently to pollen rehydration (Speranza et al., 2012) and seem to be essential for pollen apical elongation (Potocky et al., 2007) and then upon fertilization (Kaya et al., 2015).

The activity of plant Rboh proteins has been shown to be highly regulated by multiple factors. Diverse studies have described specific regulatory mechanism involving the N-terminus of these proteins (Suzuki et al., 2011), such as the activation by Ca^2+^ (Takeda et al., 2008) and phosphorylation in a synergistic way (Ogasawara et al., 2008; Drerup et al., 2013). Also, small GTPases from the Rop family and phosphatidic acid (PA) have been shown to stimulate NADPH oxidase production of ROS (Ono et al., 2001; Zhang et al., 2009). These regulatory interactions have been progressively confirmed for pollen Rbohs as well (Potocky et al., 2012; Boisson-Dernier et al., 2013; Kaya et al., 2014; Lassig et al., 2014; Jimenez-Quesada et al., 2016).

Recent studies point to RbohH and RbohJ as the source for most ROS produced at the pollen tube apex in *Arabidopsis*, since the corresponding double mutant shows defective *in vivo* and *in vitro* ROS generation (Boisson-Dernier et al., 2013; Kaya et al., 2014). However, different species seem to show distinct patterns for ROS production. Thus, the generation of ROS in lily (*Lilium formosanum*) pollen tubes was previously proposed to have a mitochondrial origin, showing an alternative localization in the subapical zone, instead of at the tip (Cardenas et al., 2006). In the cucumber (*Cucumis sativus* cv. Marketer) pollen tube, ROS are first detected at the tip but progressively become extended to the entire tube (Sirova et al., 2011). Superoxide production in kiwifruit pollen does not exhibit a clear tip localization (Speranza et al., 2012). In the growing pollen tubes of *Picea meyeri*, different ROS sources—mitochondria in the pollen tube shank and NADPH oxidase at the apex—are considered (Liu et al., 2009). Concerning their subcellular localization, and besides the expected occurrence in the plasma membrane associated to lipid microdomain (Liu et al., 2009), pollen Rbohs have been also localized at the cytoplasm (Lassig et al., 2014). Wide differences in timing, developmental patterns, and localization of ROS also occur in the stigma of numerous plant species (Zafra et al., 2016).

The study of *RbohH/RbohJ* double mutants of *Arabidopsis* suggests that RbohH and RbohJ modulate and stabilize pollen tube growth and are involved in maintenance of cell-wall integrity (Boisson-Dernier et al., 2013; Kaya et al., 2014; Lassig et al., 2014). Also, the involvement of flavoenzymes others than Rbohs in pollen tube growth cannot be excluded (Lassig et al., 2014).

Most studies carried out to date in pollen have been performed in model plants such as *Arabidopsis*, tobacco, or lily with a variety of results. This raises the question about how NADPH oxidase-produced ROS behave in pollen from other species, like the agronomically important and allergy relevant species *Olea europaea* L. (olive tree). Olive pollination is preferentially allogamous, and this plant shows genotypes with different degrees of self-incompatibility (SI), which is likely of the gametophytic type, although the precise mechanism underlying this system remains unclear (Mookerjee et al., 2005; Díaz et al., 2006; Vuletin Selak et al., 2014). Previously, ROS have been involved in gametophytic SI systems in *Angiosperms* (McInnis et al., 2006). The production of ROS and NO has also been analyzed in olive reproductive tissues along floral development (Zafra et al., 2010). However, the biochemical and molecular pathways governing and regulating the specific production of superoxide in the reproductive tissues of this plant have not been yet determined.

In this paper, we characterize the ROS-producing activity in olive pollen. For this purpose, we have cloned an *Rboh*-type gene from olive, designated as *OeRbohH*. It is mainly expressed in pollen, both during the ontogeny and the subsequent germination and tube growth. OeRbohH is a plasma membrane protein of the pollen tip. The use of specific NADPH oxidase inhibitors as well as antisense oligodeoxynucleotides (ODNs) to manage gene knockdown have demonstrated that OeRbohH is probably a tightly regulated protein required for ROS production in olive pollen. Furthermore, this ROS-producing OeRbohH activity has been revealed to be crucial for olive pollen germination and tube growth.

## Materials and Methods

### Plant Material and Growing Conditions

*Olea europaea* L. plant material was collected from selected olive trees of the cultivar “Picual,” located at the Estación Experimental del Zaidín (CSIC, Granada, Spain) or from 2-week *in vitro* germinated plantlets from embryos of the same cultivar, according to Zienkiewicz et al. (2011a). Olive pollen samples were collected during anthesis in large paper bags by vigorously shaking the inflorescences and were sequentially sieved through 150- and 50-μm mesh nylon filters to eliminate debris. All biological samples were immediately used or stored at −80°C. Tobacco (*Nicotiana tabacum*) pollen was collected as previously described (Potocky et al., 2003).

Olive pollen *in vitro* germination was initiated by a pre-hydration step in a humid chamber at room temperature for 30 min. Pollen was then transferred to the germination medium (10% [w/v] sucrose, 0.03% [w/v] Ca[NO_3_]_2_, 0.01% [w/v] KNO_3_, 0.02% [w/v] MgSO_4_, and 0.03% [w/v] boric acid), as described previously (M’rani-Alaoui et al., 2002). Pollen was maintained at room temperature in the dark, and samples were taken after hydration and at different times of germination (5 min; 1, 2, 4, 6, and 8 h). Germinated and non-germinated pollen grains from each sample were separated by filtration through 50-μm and 20-μm meshes. Tobacco pollen was *in vitro* germinated according to Kost et al. (1998).

The NADPH oxidase inhibitor diphenylene iodonium chloride (DPI, Sigma) was added to the germination medium when indicated (50 µM final concentration stocked in 2% DMSO) either at the beginning of the process (to study germination inhibition) or when pollen grain had already germinated, that is, at the beginning of third hour of *in vitro* germination (to analyze the effect on the elongation). Negative control samples were treated with DMSO, in the same proportion (2% v/v). Pollen tube length was measured using ImageJ software (http://rsb.info.nih.gov/ij/).

### Molecular Biology

#### Determination of OeRbohH Sequences

Total RNA was obtained from olive mature pollen (untreated pollen obtained immediately after anther dehiscence) using the RNeasy Plant Total RNA Kit (Qiagen). cDNA was synthesized with 1 μg of total RNA, oligo(dT)_19_ primer and M-MLV reverse transcriptase (Fermentas), according to the manufacturer’s instructions. Taq (BioTools) or Pfu (Promega) polymerases were used for PCR amplification purposes. RACE 3’ and 5’ were performed following manufacturer’s specifications (SMARTer RACE, Clontech).

#### Gene Expression Analysis

Semi-quantitative PCRs for gene expression analysis were performed using specific primers designed at the 3′-untranslated region, with Oe18S as housekeeping gene. The number of cycles was optimized for both genes to avoid reactive depletion. For quantitative PCR (qPCR), total RNA was reverse transcribed using Transcriptor High Fidelity cDNA Synthesis Kit (Roche) according to manufacturer’s instruction. Specific primers for qPCR were designed using the Primer3 software (http://frodo.wi.mit.edu/primer3/). Samples were prepared according to the LightCycler 480 SYBR Green I Master protocol, and a LightCycler 480 Instrument (Roche) was used for quantification. Relative gene expression was monitored and quantified after normalization with actin expression as the internal control. Fold variation over a calibrator was calculated using a method with kinetic PCR efficiency correction (Pfaffl, 2001), operating the relative expression ratio R = (E_target_)^ΔCP target (control−sample)^/(E_ref_)^ΔCP ref (control−sample)^, where *E* is the efficiency of target or reference amplification, and CP is the cycle number at the target or reference detection threshold (crossing point). PCR efficiencies were estimated from the calibration curves generated from the serial dilution of cDNAs.

#### Obtaining Upstream Regulatory Sequences

Genomic DNA was obtained using a NucleoSpin Tissue Kit (Macherey-Nagel). The upstream regulatory sequences were obtained by PCR walking, using an olive genomic DNA library generated according to Devic et al. (1997) as the template. Olive pollen genomic DNA libraries were generated by digestion with restriction enzymes (DraI, EcoRV, PvuII, ScaII, SspI, StuI, and HpaI) and ligation of known sequence adaptors in both extremes. MBLong polymerase (MOLBIOLAB) was used. A fragment of 1.8 kb from the start codon of *OeRbohH* was obtained.

#### Construction of the OeRbohH Promoter-β-Glucuronidase (GUS) Fusion

The fragment of 1.8-kb upstream the start codon of *OeRbohH* was amplified with specific primers incorporating a restriction site (**Supplementary Table 1**) to allow cloning into the binary vector pBI101.1 (Clontech) at the initiation codon of the promoterless *GUS* gene.

#### Construction of OeRbohH-GFP and OeRbohH-YFP Fusions

YFP fusion proteins were constructed in vectors pWen240 for (N-terminal fusion) and pHD32 (C-terminal fusion), under the control of the pollen-specific Lat52 promoter. Sequences were obtained using the specific forward oligonucleotide OeRboh-Ngo-F and the reverse oligonucleotides OeRboh-Xma-RS or OeRboh-Xma-RNS, to incorporate the appropriate restriction site.

#### Construction of OeRbohH Expression Vector

For open reading frame (ORF) cloning purpose, 2,502-bp-long cDNA of OeRbohH was cloned into the expression vector pET51b+ expression vector (Novagen). The sequence was amplified using the specific forward oligonucleotide OeXbaRbohF and the reverse OeSacRbohR, to incorporate in the appropriate restriction site (XbaI/SacI).

All primers used in this work are described in **Supplemental Data, Table S1**.

### Biochemistry

#### Protein Extracts

For protein extraction, pollen was powdered in liquid nitrogen and re-suspended in extraction buffer [50 mM phosphate buffer (pH 7.8), 1mM PMSF] to a proportion of 15 ml solution per gram of fresh tissue, and proteins were eluted by stirring for 2 h at 4°C. After centrifugation at 13,000 ×g for 20min at 4°C, the supernatants were filtered through a 0.22-μm mesh and used for activity assays and Western blot analysis. The protein concentration in each sample was measured following the Bradford (1976) method, using the Bio-Rad reagent and bovine serum albumin (BSA) as standard.

#### Native PAGE and In-Gel NADPH Oxidase Activity Assay

Samples prepared as above were loaded into a 7.5% native acrylamide gel (80 μg protein per lane) as described by Davis and Ornstein (1964). Proteins were separated using a Mini-PROTEAN II system (Bio-Rad, U.S.A.).

The presence of NADPH-dependent production of O_2_^•−^ was tested in gels by the NBT (Nitroblue tetrazolium) reduction method as previously described (Potocky et al., 2007). The gel was incubated in the developing solution [50mM Tris-HCL (pH 7.4), 0.2mM MgCl2, 1mM CaCl_2_, 0.5 mg/ml NBT (Sigma), and 0.2 mM NADPH (Sigma)] and incubated at RT overnight under gentle shaking. The reaction was stopped by immersing the gel in distilled water. To test the effects of specific inhibitors, DPI (50 μM) and sodium azide (10 mM) were added to the developing solution.

#### *In Vitro* Expression of OeRbohH in *Escherichia coli* and Rise of an OeRbohH Antibody

Recombinant protein carrying a 10-His tag in N-terminus was over-expressed in *Escherichia coli* strain *BL21 Rosetta 2* (Novagen) according to Traverso et al. (2013a). An overnight pre-inoculum (1ml) was added to LB medium supplemented with 50 mg/ml of ampicillin and 34 mg/ml of chloramphenicol and incubated at 21–22°C until OD_600_ of 0.6 was reached. Expression was then induced with 0.4 mM isopropylthio-b-galactoside (IPTG), and cells were incubated for another 12 h. Finally, cells were harvested by centrifugation. Crude protein extracts corresponding to the disrupted bacteria, both from the expression construct and the negative control, were subjected to SDS-PAGE analysis. Differential bands were excised, digested with trypsin, and subjected to LC-ESI-MS/MS in the proteomic facilities of CNB-CSIC (Madrid, Spain). Mass data collected were used to search using a local Mascot server (Matrix Science, London, UK) against an in-house-generated protein database composed of protein sequences of *Viridiplantae*. Carbamidomethylation of cysteine (+57Da) and oxidation of methionine (+16Da) were set as variable modifications. Protein identification was confirmed using a Mascot’s threshold score of identity at a 95% confidence level.

One of the fragments (corresponding to a 35-kDa band) was used to immunize hens in order to obtain a polyclonal antibody against OeRbohH (Davids Biotechnologie GmbH).

#### Immunoblotting of OeRbohH

Total protein per sample was loaded on 12% (w/v) polyacrylamide gels and electrophoresed using a Mini-PROTEAN 3 apparatus (Bio-Rad). After electrophoresis, proteins were electroblotted onto a polyvinylidene fluoride (PVDF) membrane in a Semi-Dry Transfer Cell (Bio-Rad) at 100V during 1h. For immunodetection, membranes were incubated in blocking buffer [3% (w/v) milk in Tris-buffered saline (TBS) buffer] at RT during 1 h, followed by incubation in primary antibody (anti-OeRbohH), diluted 1:1,000 in TBS buffer (pH 7.4) containing 0.1% (v/v) Tween-20 overnight at 4°C under shaking. Incubation in secondary antibody (1:2,000 dilution) conjugated to Alexa Fluor 488 (Invitrogen) was performed at RT during 1 h under agitation. The fluorescent signal was detected in a Pharos FX Molecular Imager (Bio-Rad).

### Nucleic Acid Transfection

#### *Arabidopsis thaliana* Transformation and GUS Assays

Binary constructions were introduced into *Agrobacterium tumefaciens* (C58pMP90). *Arabidopsis* (*Arabidopsis thaliana*; ecotype Columbia) was transfected by the floral dip method (Clough and Bent, 1998). T1, T2, and T3 seedlings were selected *in vitro* on Murashige and Skoog medium supplemented with 1% (w/v) sucrose, 0.8% (w/v) agar, and 30 *μ*g/ml kanamycin under a 16-h-light/8-h-dark regime at 22°C. Plants were cultivated in soil and under the same conditions as described above. GUS histochemical staining was performed according to Jefferson et al. (1987).

#### Biolistic Transformation

Germinated pollen grains from olive and tobacco were bombarded with gold particles alternatively coated with two different in-frame constructs of OeRbohH and the fluorescent yellow protein (YFP either at the amino- or the caboxi-terminus of the construct) and with the YFP alone (control), using the PDS-1000/He instrument (Bio-Rad) as previously described (Traverso et al., 2013a; Potocký et al., 2014).

#### Design of Antisense Oligodeoxynucleotides (ODNs) and Delivery Into Growing Pollen Tubes

*OeRbohH* sequence was tested for accessibility prediction and effective design of antisense ODNs with Soligo software (http://sfold.wadsworth.org/cgi-bin/soligo.pl). Proposed antisense and corresponding sense control ODNs were synthesized with phosphorothioate modifications in both the 5′- and 3′ terminus. Three antisense/sense ODNs pairs were essayed, and the most effective pair was selected (antisense: TAAGCAATCTTCGCCTGGTG; sense: CACCAGGCGAAGATTGCTTA). For the transfection, ODN-cytofectin complexes were prepared as described previously (Moutinho et al., 2001; Bezvoda et al., 2014) and incorporated to the germination medium. Control samples were incubated as described; however, no ODNs were used.

### Microscopy Analyses

#### Immunocytochemistry

For immunocytochemistry, fresh anthers at different stages of development were prepared as described previously (Zienkiewicz et al., 2011b). Germinated pollen samples were fixed, and tube walls were digested as described before (Zienkiewicz et al., 2010). Slides containing semi-thin sections of olive anthers at different developmental stages and germinated pollen samples were incubated in blocking solution containing 1% (w/v) BSA in phosphate buffered saline (PBS) solution (pH 7.2) for 1 h at room temperature. OeRbohH was immunodetected by incubation of slides overnight at 4°C with the anti-OeRbohH primary Ab (diluted 1:10 in blocking solution) followed by an anti-chicken IgG-Alexa Fluor 488–conjugated secondary antibody (Ab) (Molecular Probes, USA) (diluted 1:100 in blocking solution) for 1 h at 37°C. In control sections, the preimmune serum was used. A few drops of an anti-fading solution of Citifluor (Sigma-Aldrich) were added, and samples were observed with a Zeiss Axioplan epifluorescence microscope under blue light irradiation.

#### Detection of O_2_^•−^ Production

Detection of O_2_^•−^ production in growing pollen tubes was determined by incubation in growth medium containing 1 mg ml^−1^ NBT for 5 min. Negative control experiments were carried out the same; however, no NBT was added to the growth medium. Inhibition experiments were performed by incubating pollen tubes in growth medium containing 50 μM DPI (30 min) before NBT staining. Alternatively, the effect of sodium azide (10 mM) was also tested. Samples were observed with a Zeiss Axioplan microscope under bright field. Quantification of the intensity of the dark purple-colored precipitate was performed in the pollen tubes through 50 µm along the apical region of the pollen tubes starting at the pollen tube apex. Quantification was achieved by using the Nikon EZ-C1 viewer (3.30) software, selecting the region of interest (ROI) and analyzing pixel intensity, which was referred to the area of the ROI, therefore calculating the ratio color intensity/area in arbitrary units. Both average and standard deviation were calculated after measurement of a minimum of 10 images of pollen tubes per experiment, along three independent experiments.

### Bioinformatic Tools

Protein amino acid sequences were aligned using Clustal Omega multiple alignment tool with default parameters (McWilliam et al., 2013). Phylogenetic trees were constructed with the aid of the software SeaView (Gouy et al., 2010) using the maximum likelihood (PhyML) method and implementing the most probable amino acid substitution model (LG) previously calculated by the ProtTest 2.4 server (Darriba et al., 2011). The branch support was estimated by bootstrap resampling with 100 replications. OeRbohH motifs were predictively analyzed by the software “PredictProtein” according to the proteins HsNox2 (Sumimoto, 2008; von Lohneysen et al., 2010) and OsRbohB (Oda et al., 2010).

### Statistical Analysis

Statistical significance was determined by Kruskal–Wallis one-way analysis of variance followed by Kruskal–Wallis Multiple-Comparison Z-Value Test (Dunn’s Test).

## Results

### OeRbohH Is an Rboh-Type Gene Present in the Olive Tree, Which Is Mainly Expressed in Pollen

Considering that superoxide (O_2_^•−^) generation in mature pollen is described as mainly produced by the physiological activity of NADPH oxidases (Potocky et al., 2007); we decided to investigate the occurrence of NADPH oxidases in the olive (*O. europaea* L.) tree. For such purpose, we initially obtained the sequence of an olive Rboh gene expressed in pollen. Several pairs of degenerated primers were designed on conserved domains identified in other species (**Supplemental Data, Figure S1**). Using primers OeRboh804F and OeRboh2286R, we initially sequenced a 300-bp fragment. From this nucleotide sequence, two primers were designed and used to obtain the 5’ and 3’UTR (untranslated) sequences by RACE (rapid amplification of cDNA ends) (OeRbohRW2 and OeRbohFW2, respectively). This enabled us to obtain the whole coding sequence (CDS) of 2,502bp (GenBank KX648357).

**Figure 1:**
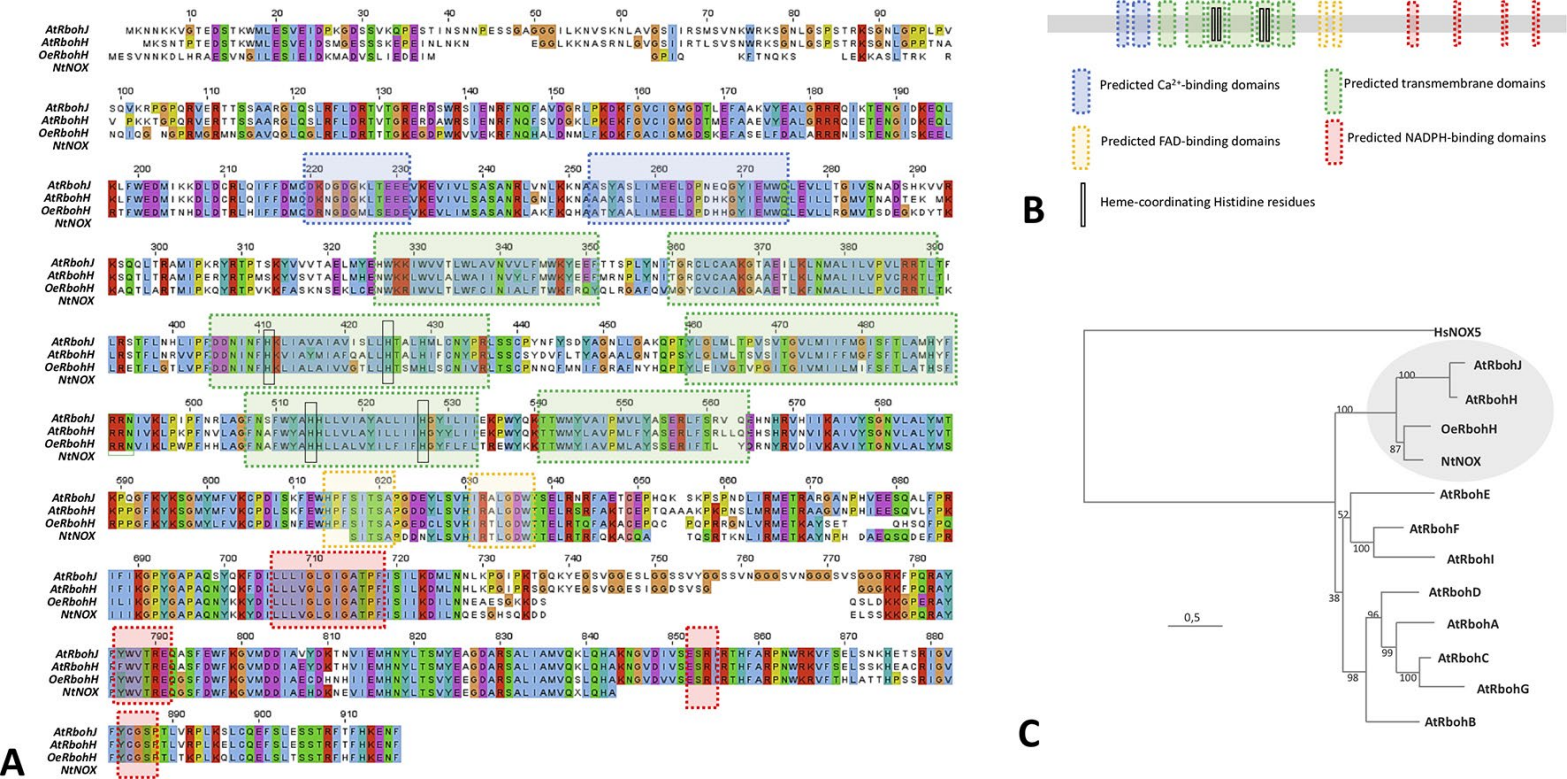
**(A)** Amino acid sequence alignment and predicted motifs of OeRbohH, AtRbohH, AtRbohJ, and NtNOX. Conserved residues are highlighted in red. The blue boxes indicate the predicted calcium-binding domains (2). Predicted transmembrane domains (6) are boxed in green, including the conserved heme-coordinating histidine residues (4) highlighted with black circles. The predicted FAD-binding sites are marked with purple boxes (2). Predicted sites for NADPH binding (4) are indicated with red boxes. **(B)** Analogous color coding was used to show domains/motifs in a simplified diagram of OeRbohH. **(C)** Phylogenetic relationships of the 10 NADPH oxidase proteins from *Arabidopsis* (AtRbohA-J), together with olive pollen OeRbohH and a partial tobacco pollen sequence Rboh NtNOX. The tree was constructed by the maximum likelihood (ML) from Clustal Omega multiple alignment and rooted with human NOX isoform HsNOX5. Numbers at nodes indicate bootstrap values. The circle marks the putative pollen-Rboh subgroup.

The corresponding translated amino acid sequence was denominated OeRbohH according to its identity with AtRbohH (62.11%), which was higher than that displayed with AtRbohJ (60.77%). It also contained all the characteristics structural and catalytic motifs to be considered as a plant Rboh (**Figures 1A, B**). Secondary structural motifs and domains were identified *in silico* accordingly to the proteins HsNox2 (Sumimoto, 2008; von Lohneysen et al., 2010) and OsRbohB (Oda et al., 2010), and the predictive software “PredictProtein.” Thus, OeRbohH enzyme was predicted to contain six α-helix transmembrane domains—which included the conserved heme-coordinating His residues—together with two terminal cytosolic extensions. The C-terminus contained four sites for NADPH binding and two sites for FAD binding, and the N-terminal end was predicted to contain two EF-hand Ca^2+^-binding motifs (**Figures 1A, B**).

Moreover, a phylogenetic analysis among OeRbohH and other available plant Rboh sequences showed OeRbohH clustered in a subgroup containing the pollen specific Rbohs from *A. thaliana* and tobacco (**Figure 1C**), which are the unique pollen-specific Rbohs identified so far (Potocky et al., 2007; Boisson-Dernier et al., 2013). In addition, primers OeRbohF and OeRbohR were designed and used to amplify the genomic sequence (GenBank KX648358) corresponding to the whole ORF (4,025 bp). Alignment between the obtained genomic sequence and the corresponding ORF allowed us to identify 13 introns (**Supplemental Data, Figure S2**) whose positions relative to the ORF were conserved within the plant Rboh family (**Supplemental Data, Figure S3**).

**Figure 2:**
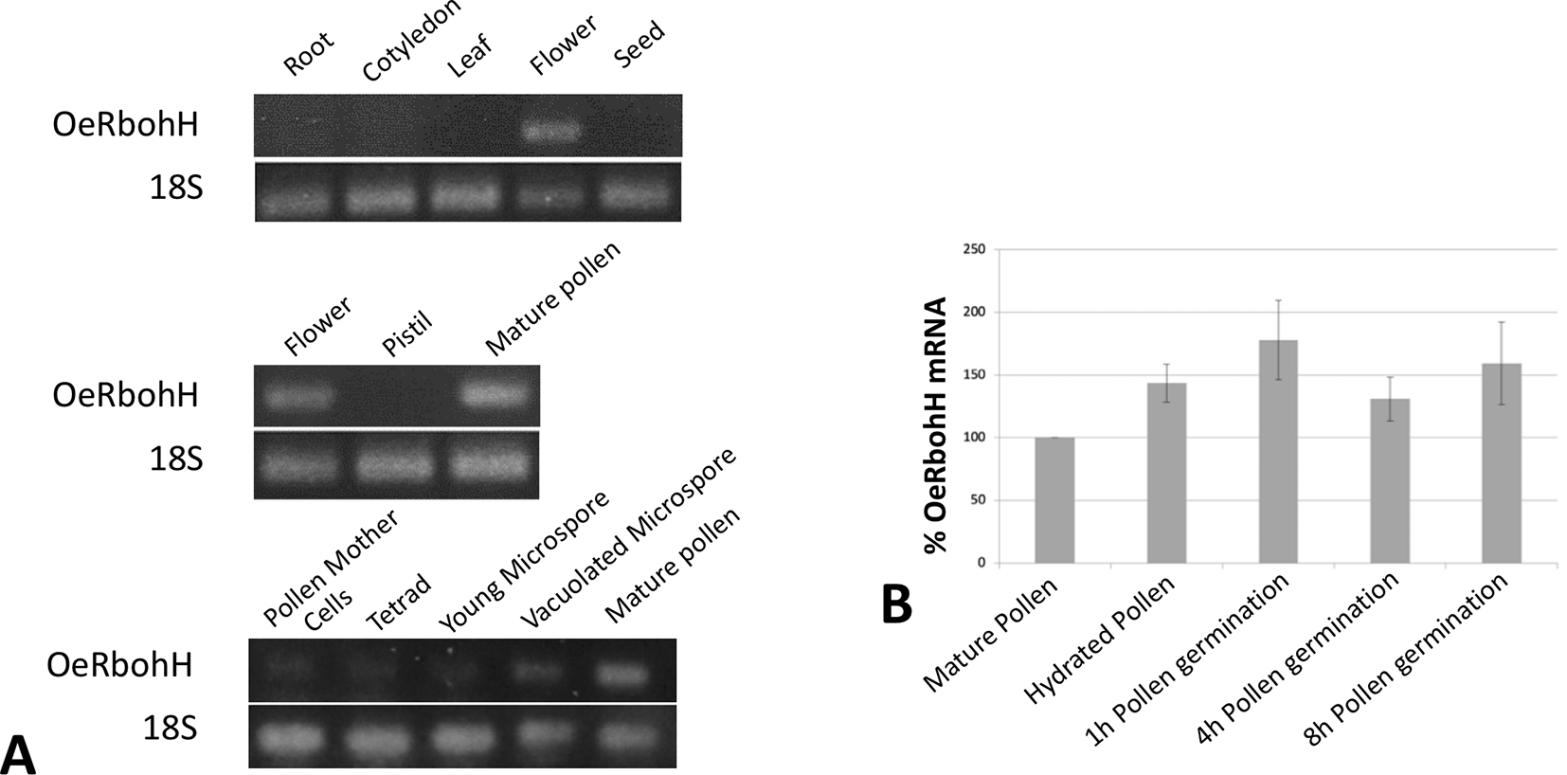
**(A)** OeRbohH expression analysis using semiquantitative PCR of seedling and tree tissues (upper panel), floral tissues (medium panel), and pollen ontogeny (lower panel). **(B)** mRNA levels of OeRboh determined by real-time PCR in pollen along *in vitro* germination, expressed as percentages compared to the mRNA level of the mature pollen (100%). Averages of three biological replicates ± SD are presented. No significant differences were found. ANOVA test (p < 0.05).

With the purpose of studying gene expression pattern of OeRbohH, a pair of primers was designed in the 3’UTR region of OeRbohH, and cDNAs from different vegetative and reproductive organs were synthesized to be used as template. Semiquantitative PCRs were carried out in a first approach, and OeRbohH expression was almost exclusively detected in flowers (**Figure 2A**, see discussion). Within the flower, expression was detected in pollen (**Figure 2A**) and not in the gynoecium.

We also measured the gene expression level of OeRbohH throughout pollen ontogeny by using cDNA from anthers at different stages. OeRbohH was expressed both during microsporogenesis (pollen mother cells to the tetrad stage) and microgametogenesis (**Figure 2A**). Evidently, this result does not allow us to discriminate whether the expression of OeRbohH in the anther takes place in the sporophytic or the gametophytic tissues. In addition, taking into account that pollen Rbohs has been suggested to be involved in pollen tube growth (Kaya et al., 2014; Lassig et al., 2014); qPCR analysis was performed in the course of pollen *in vitro* germination. Variations of gene expression at these stages would differently involve OeRbohH in different aspects of pollen physiology. However, the expression level of OeRbohH during pollen tube growth was rather stable, with a relative, although not statistically, significant peak of expression at 1 h of *in vitro* germination (**Figure 2B**).

In order to improve our knowledge about OeRbohH, we also decided to analyze the expression pattern of the reporter gene β-glucuronidase (GUS; UidA) under the control of a 1.8-Kb fragment from the upstream regulatory sequences of OeRbohH in *Arabidopsis thaliana* transgenic lines. However, in these lines, we were unable to detect UidA expression (data not shown), which is in good agreement with the low expression level found during the semi-quantitative analyses (see discussion).

### Bacterial Expression of OeRbohH and Determination of the Specificity of the Risen Antibody

*In vitro* expression of OeRbohH in *E. coli* as a recombinant protein tagged with a multiple His was monitored by SDS-PAGE. The *E. coli* strain expressed OeRbohH efficiently, although the extracts of the induced cultures displayed three differential bands of c.a. 100, 80, and 35 kDa in the insoluble fraction, the latter showing reactivity to an anti-His Tag antibody in further Western blotting experiments (**Supplemental Data, Figure S4, A, B**).

The three bands were excised from gels and subjected to mass spectrometry (MS) analysis. They were identified by LC-ESI-MS/MS (liquid chromatography–electroSpray ionization–tandem mass spectrometry) as different fragments corresponding to translated product of OeRbohH (**Supplemental Data, Table S2**). Fragment 3 was used to obtain the antibody (see material and methods).

The binding specificity of the anti-OeRbohH antibody developed in hen was tested by immunoblotting (**Supplemental Data, Figure S4, C**). The antibody recognizes a single band in the membranes resulting from the transference of native gels with electrophoresed extracts of both mature pollen and pollen during *in vitro* germination. These bands were coincident in electrophoretic mobility with the activity bands revealed by in-gel NADPH oxidase activity assay (**Supplemental Data, Figure S4, D**).

**Figure 3:**
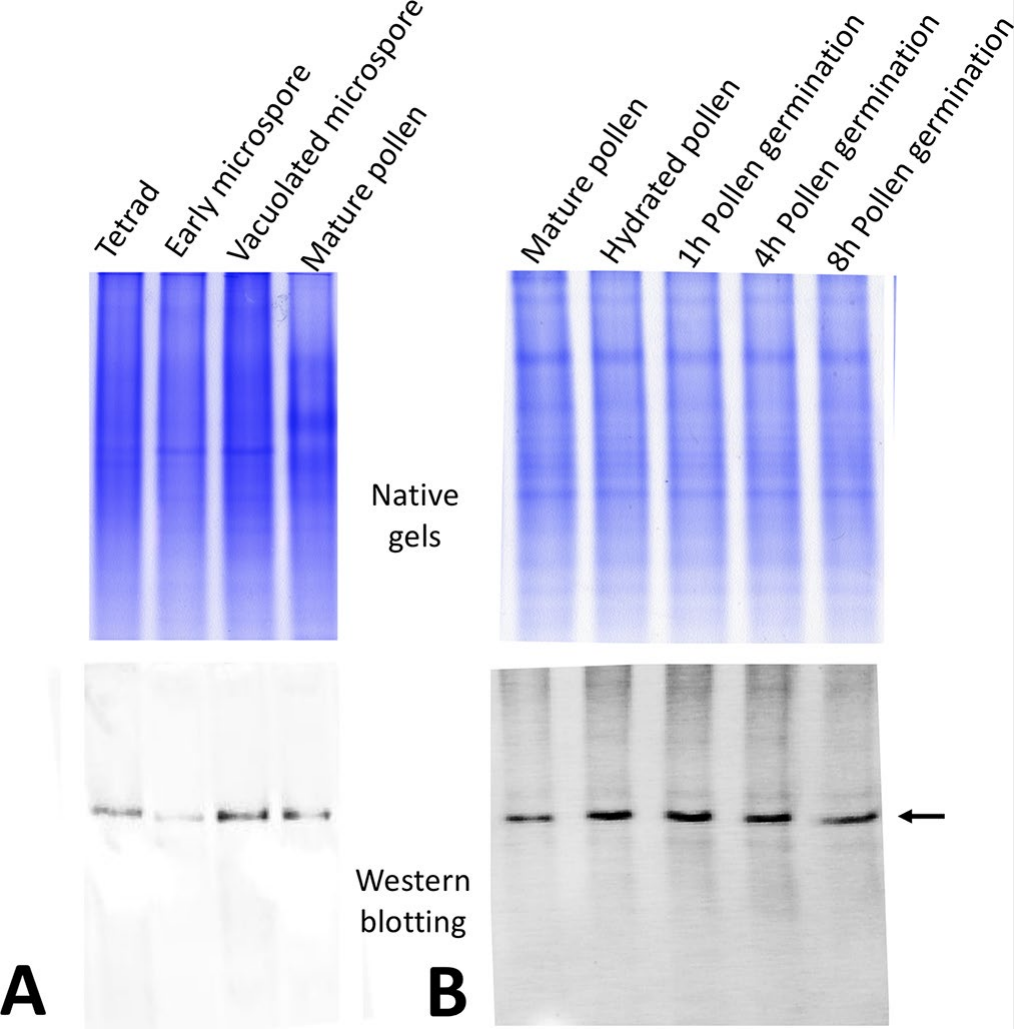
OeRbohH protein expression along pollen ontogeny **(A)** and *in vitro* pollen germination **(B)**. Western blot using the anti-OeRbohH antibody (lower panel) was performed after native-PAGE (upper panel).

Using this anti-OeRbohH antibody, we performed the immunoanalysis to investigate the protein expression during pollen ontogeny and *in vitro* pollen germination (**Figure 3A**). One band was detected in all of the developmental stages analyzed, although cross-reactivity with other Rboh isoforms expressed in anthers could not be rejected. We then probed pollen protein extracts at different times after the onset of the *in vitro* germination, and a single band was detected during the process (**Figure 3B**).

### OeRbohH Protein Localizes in Both the Developing Pollen Grains and the Sporophytic Tissues of the Anther

**Figure 4:**
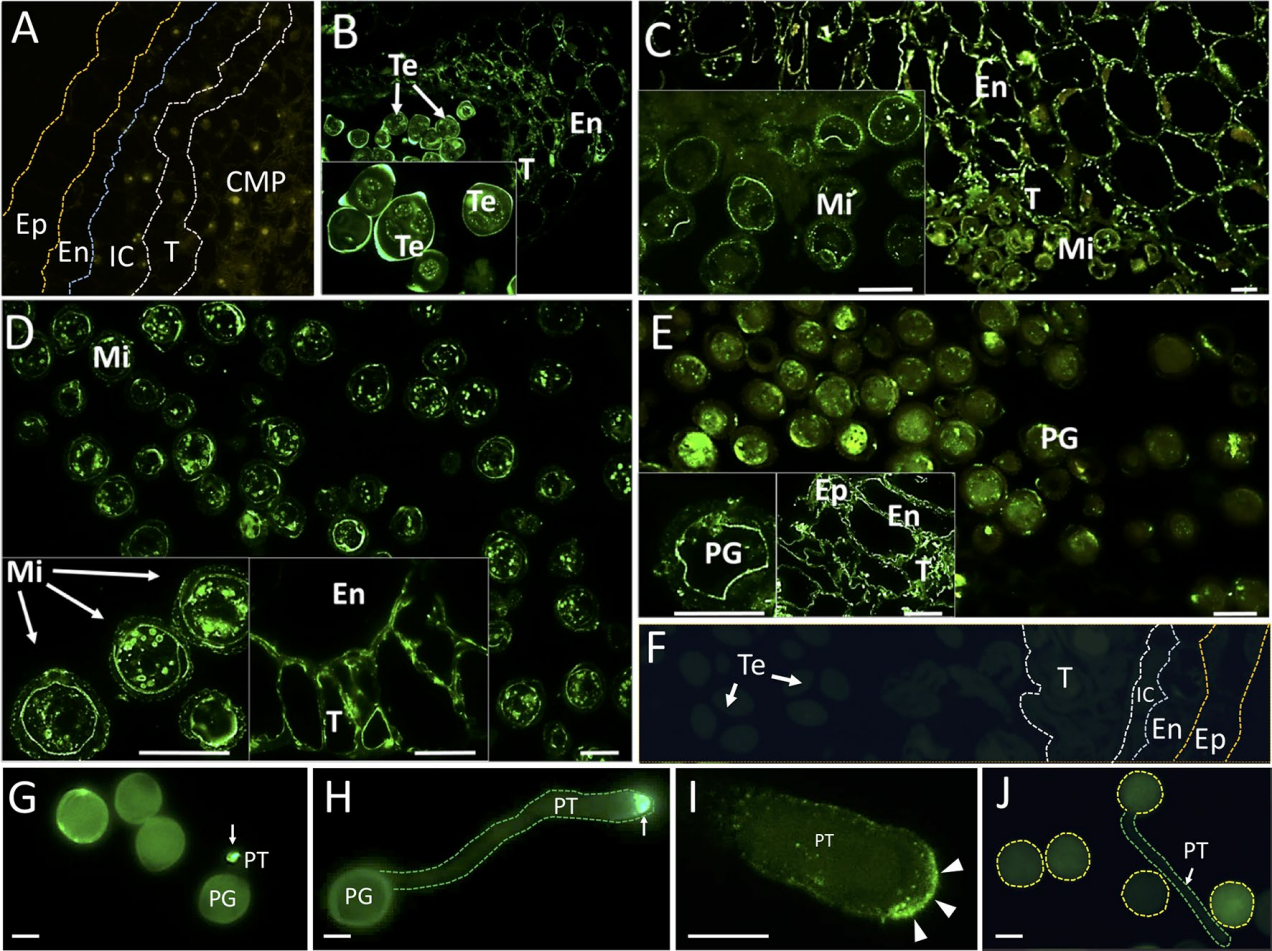
Fluorescence microscopy localization of OeRbohH in the olive anther and *in vitro* germinated pollen grains. Sections from olive anthers at the following stages: pollen mother cells prior to meiosis **(A)**, tetrads **(B)**, young microspores **(C)**, vacuolated microspores **(D)**, and mature pollen **(E)** were incubated with an anti-OeRbohH Ab, followed by an anti-chicken IgG-Alexa Fluor 488–conjugated secondary Ab. In insets, detailed view of gametophytic tissue is shown in B-E. Right insets in D and E show a detailed view of the sporophytic tissues of the anther. Negative control sections (anthers at the tetrad stage) were treated with the preimmune serum **(F)**. *In vitro* germinated pollen grains were also used for fluorescence microscopy localization of OeRbohH. Recently emerged pollen tube showed intense labeling at the pollen tube tip (**G**, arrow). Elongated pollen tubes also showed intense labeling at the tip (**H**, arrow). High magnification of the pollen tube apex after immunolocalization of OeRbohH **(I)**. The protein accumulates at the very tip, although it can be also weakly localized at the plasma membrane (arrowheads). Negative control (using the preimmune serum as the primary antibody) did not show labeling **(J)**. Note the autofluorescence of the exine. En, endothecium; Ep, epidermis; IC, intermediate cells; Mi, microspore; MP, mature pollen grain; T, tapetum; Te, tetrad; YP, young pollen. Bar = 20µm. Boundaries of the anther layers and the pollen grain/pollen tube contour are shown for reference in several pictures **(A, F, H, J)**.

Immunohistochemical experiments were performed to localize OeRBOH *in situ* along pollen ontogeny. OeRbohH antibody signals were evident not only in the developing pollen grains but also in some cell types from the sporophytic surrounding tissues (**Figure 4**).

Young anthers containing pollen mother cells were barely labeled (**Figure 4A**). Anti-OeRbohH labeling was detected during microsporogenesis at spots placed in the vicinity of the plasma membrane within the tetrad. In addition, signal was likewise detected in a low level in both the anther wall layers (**Figure 4B**). These anther tissues showed a more evident OeRbohH labeling after microspore release (**Figure 4C**). OeRbohH signal was located at the plasma membrane of young free microspores (**Figure 4C**, magnification) and vacuolated microspores which were also labeled intracellularly at unidentified spots/rings (**Figure 4D**). The mature pollen grain showed signal at the plasma membrane and also at cytoplasmic spots (**Figure 4E**). During pollen maturation, fluorescence was present in both the endothecium and the tapetum remnants (**Figures 4D, E**, magnifications). Negative control sections, treated with the preimmune serum instead of the anti-OeRbohH antibody, showed no relevant fluorescent signal (**Figure 4F**).

### OeRbohH Is a Plasma Membrane Protein With Enhanced Activity at the Pollen Tip, Whose Activity Must Be Highly Regulated

OeRbohH localization during olive pollen tube growth was carried out by immunohistochemistry. *In vitro* germinated pollen displayed enhanced fluorescence at the pollen tube tip since tube emergence, and until the tube reaches high lengths (**Figure 4G–I**), in contrast with the samples subjected to the control treatment (**Figure 4J**), which displayed no signal.

**Figure 5:**
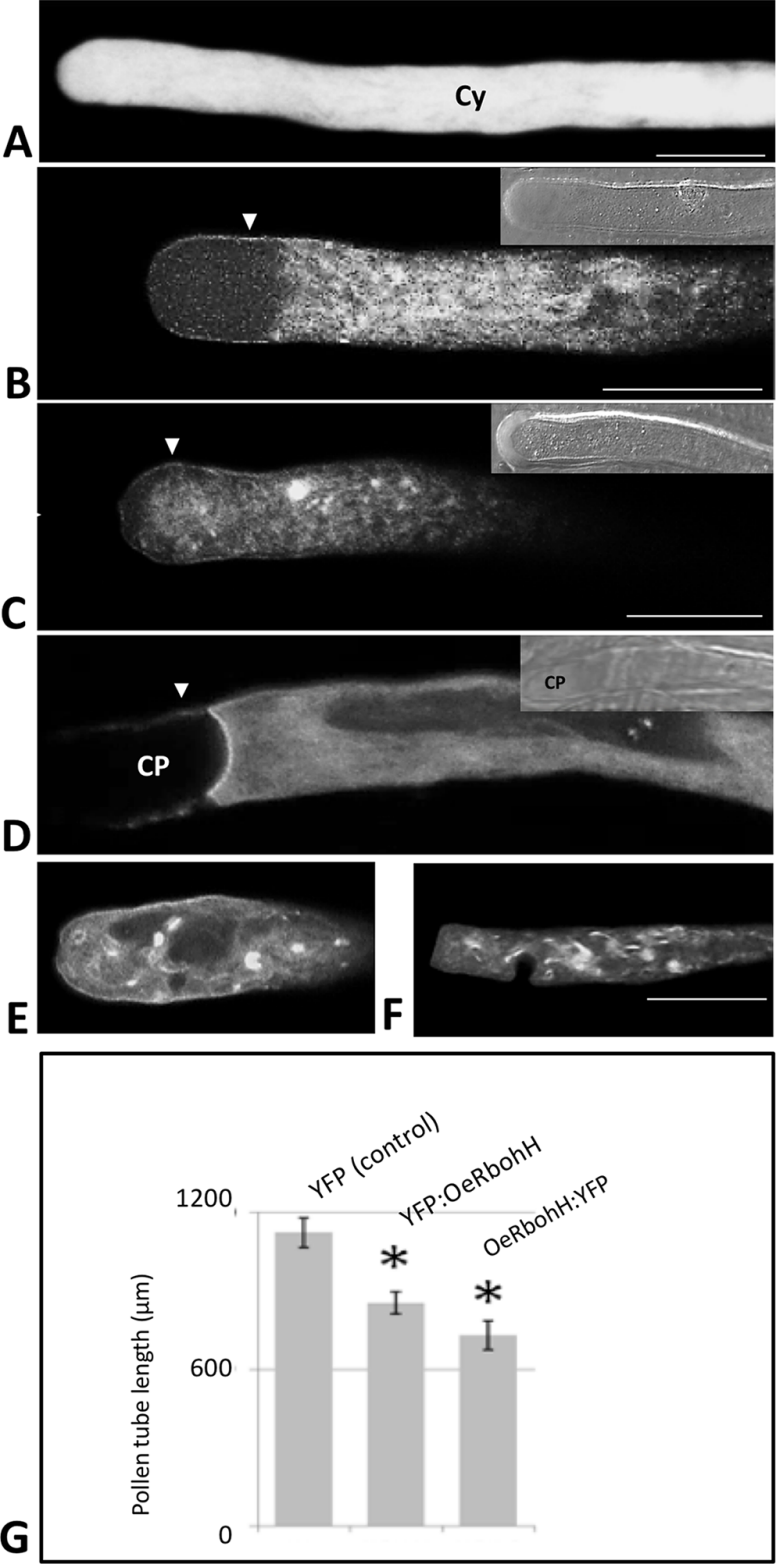
Transient expression of OeRbohH in tobacco pollen tubes as YFP fusions, observed by CLSM. **(A)** YFP construction alone (control) showed homogeneous yellow fluorescence throughout the cytoplasm of the pollen tube. **(B)** YFP : OeRbohH and **(C–F)** OeRbohH : YFP transformants showed labeling in the plasma membrane (arrowheads) as well as in the cytoplasm without significant differences among both constructs. **(D)** Fluorescence was also observed at the edge of callose plugs in their proximal side (referred to the pollen grain). The majority of transformed pollen tubes showed accumulation spots **(C, E, F)** and abnormal phenotypes (i.e., swelling) at the tip **(E–F)**, and their growth was significantly inhibited after 4h *in vitro* culture **(G)**. Insets at pictures **B**, **C**, and **D** show bright field images of the pollen tubes for reference. CP, callose plug; Cy, cytoplasm. *indicates that the mean is significantly different from the control at P < 0.05. n = 100 from three independent experiments. Bars = 20 µm.

We also used an *in vivo* strategy to analyze the subcellular localization of OeRbohH. The fluorescent chimeras OeRbohH-YFP or YFP-OeRbohH were transiently expressed in growing pollen tubes of a heterologous system (tobacco) by biolistic transformation driven by the control of the pollen-specific Lat52 promoter, whereas olive pollen transformants were barely found. Pollen tubes expressing YFP only showed homogenous fluorescence signal all-through the pollen tube cytoplasm (**Figure 5A**). Alternatively, growing pollen tubes transformed with the YFP-OeRbohH (**Figure 5B**) or OeRbohH-YFP (**Figures 5C–F**) constructs were preferentially labeled at the tip plasma membrane along with signal detected in the cytoplasm and the endomembrane system (**Figures 5B, C**), as it has been previously reported in *Arabidopsis* pollen (Boisson-Dernier et al., 2013; Lassig et al., 2014). Accumulation of fluorescence also occurred at the proximal boundary of the callose plugs (referred to the pollen grain) in the OeRbohH-YFP transformed pollen tubes (**Figure 5D**). Several morphological alterations, i.e., accumulations in the endomembrane system and presence of accumulation spots (**Figures 5E, F**), suggest a physiological unbalance due to an excess of OeRbohH in the transformants. Furthermore, pollen tube elongation was reduced in the transformant grains (**Figure 5G**).

OeRbohH is an Rboh-type protein from olive, which localizes to the plasma membrane and endomembranes in the growing pollen tube tip and whose over-expression affects pollen tube integrity and physiological growth.

### Oxidative Burst in the Olive Pollen Tube Tip Depends Mainly on NADPH Oxidase Activity

**Figure 6:**
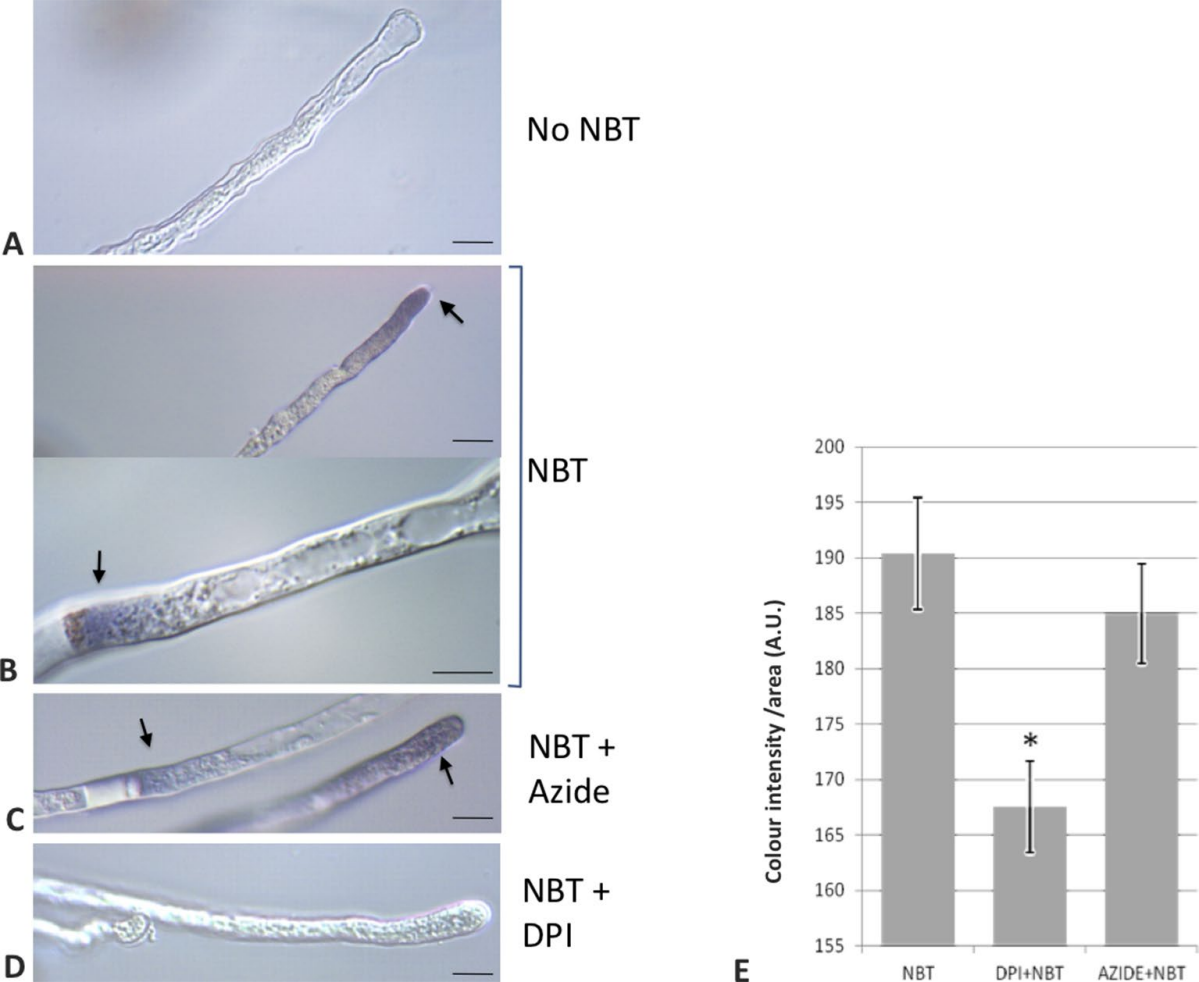
Detection of O_2_^•−^ putatively generated by NADPH oxidase activity in pollen tubes. **(A)** Control sample incubated in growth medium in the absence of NBT. **(B)** NBT precipitate is observed in the pollen tube near the apex and in the proximity of callose plugs. **(C)** NBT stain remains even in the presence of sodium azide, an inhibitor of NADPH peroxidase. **(D)** Addition of NBT in the presence of DPI, an inhibitor of NADPH oxidases, did not lead to staining. **(E)** Quantification of the precipitate intensity along 50 µm of the apical region of the pollen tubes. Data represent means ± SEM. *indicates that the mean value is significantly different from the control at P < 0.001. n = 100 from three independent experiments. Bars = 10 µm. NBT, NBT-treated samples; DPI+NBT, samples treated with NBT and DPI; AZIDE+NBT, samples treated with NBT and sodium azide; A.U., arbitrary units.

To provide empirical evidence supporting the involvement of NADPH oxidase activities during the germination of olive pollen, as it has been previously proposed for model plants such as *Arabidopsis* or tobacco (Potocky et al., 2007; Boisson-Dernier et al., 2013), a set of experiments were carried out during olive pollen *in vitro* germination. The short-lived superoxide radical was preferentially produced at the tip of the growing pollen tube as it was revealed by the NBT staining (**Figures 6A, B, D**). Occasionally, the cytoplasm in the proximity of the proximal end of the callose plugs (referred to the pollen grain) was also labeled (**Figures 6C, D**).

**Figure 7:**
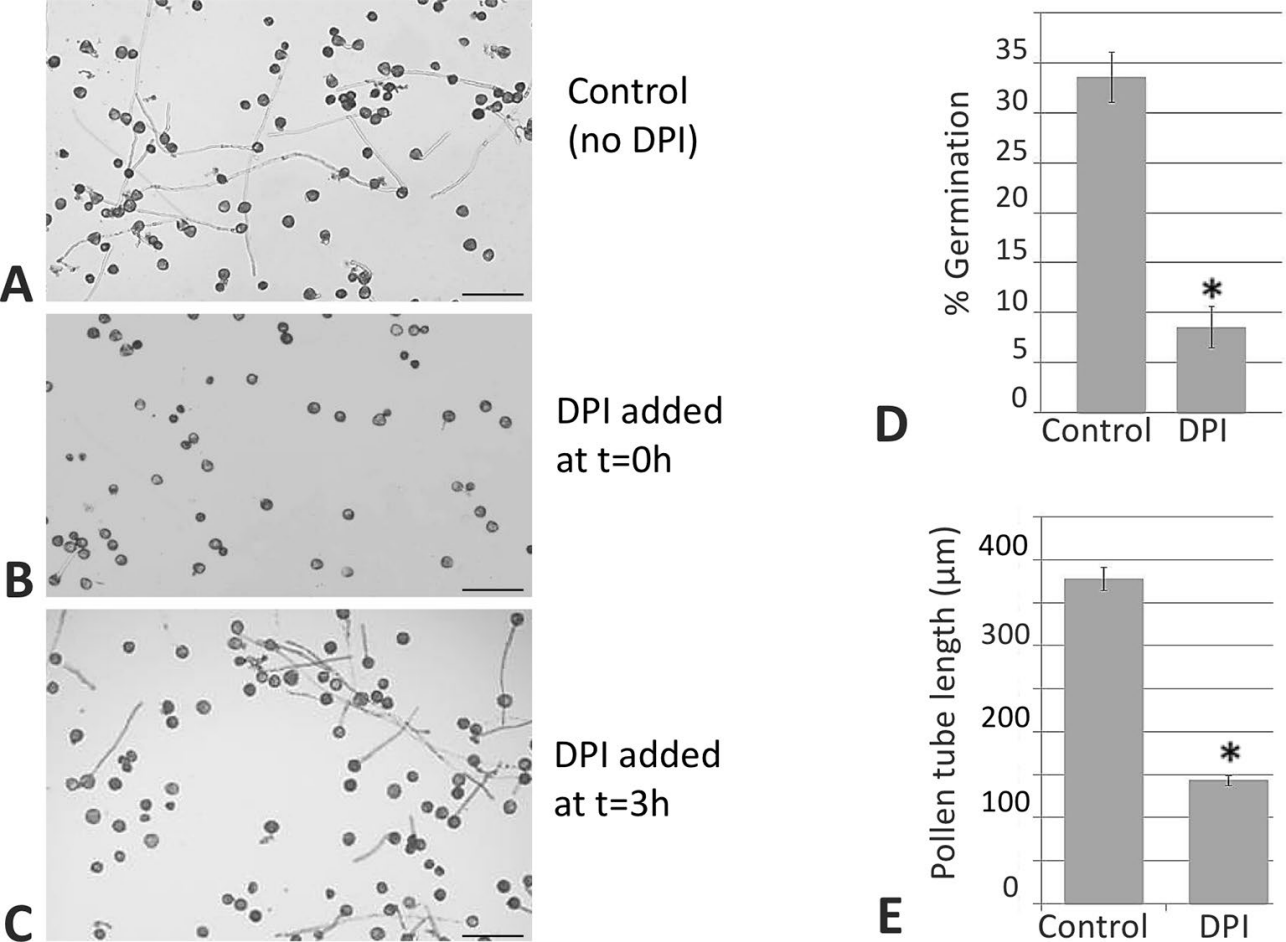
DPI inhibits germination and pollen tube elongation. Control sample **(A)**. When the NADPH oxidase inhibitor was added at the beginning of the process, the *in vitro* germination percentage was affected **(B)**. When the inhibitor was added during the germination, the length of the pollen tube was affected **(C)** in both cases when compared to the control. Representative pictures from three independent experiments are shown. Quantification of the germination rate **(D)** and pollen tube lengths **(E)**. Data represent means ± SEM. n = 100. *indicates that the mean is significantly different from the control at P < 0.05. Bars = 100 µm.

**Figure 8:**
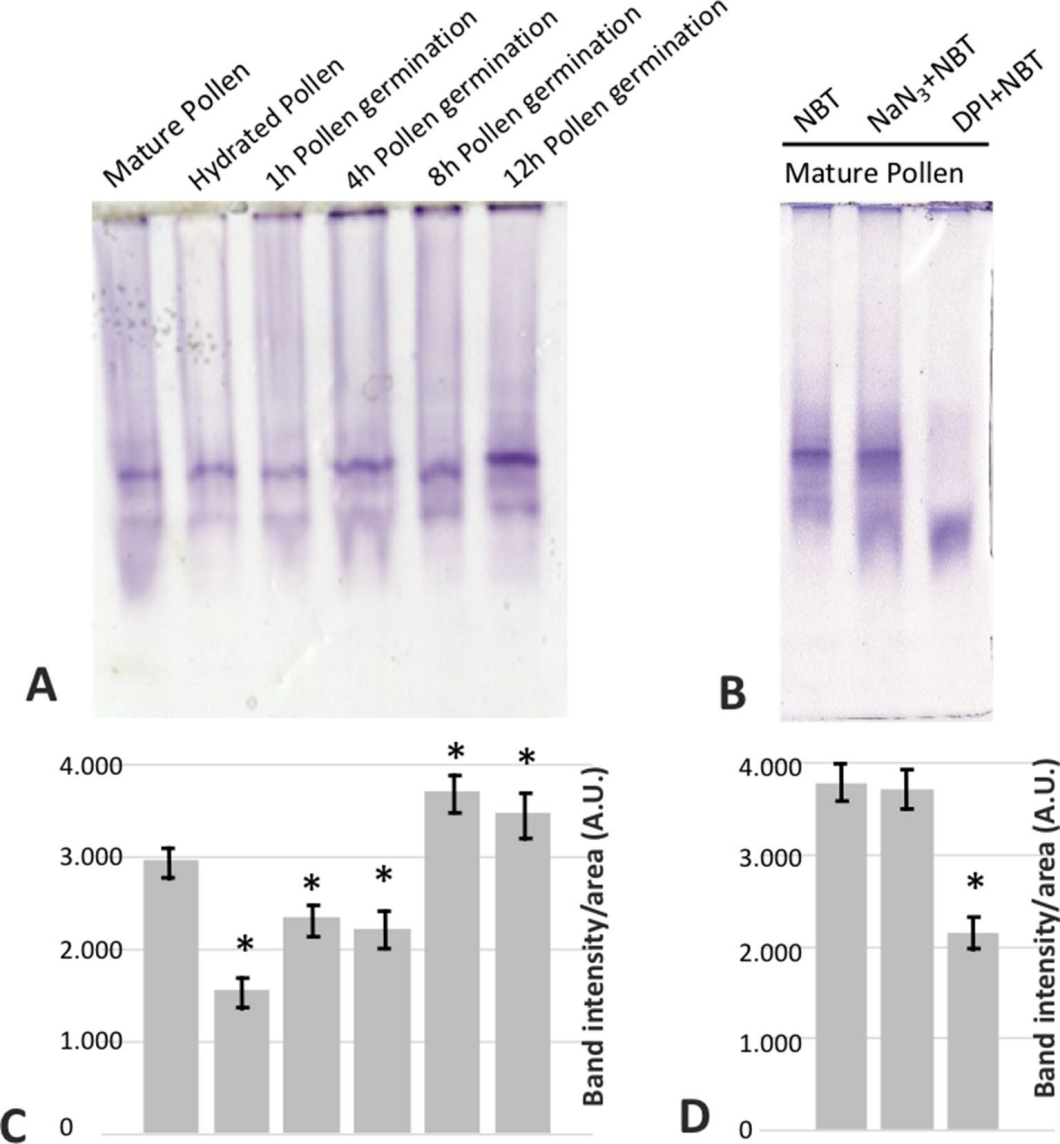
**(A)** In gel NADPH oxidase activity of mature (MP), hydrated (H), and germinated pollen extracts at different times (1, 4, 8, and 12 h) fractionated by native PAGE (50 µg/lane). **(B)** In mature pollen, the activity revealed by NBT was challenged by the addition of sodium azide or DPI during the incubation. **(C, D)** Densitometry quantification of NADPH oxidase activity as in **(A)** and **(B)**, respectively. *indicates that the mean from three independent experiments is significantly different from the control (mature pollen) at P < 0.05.

In addition, with the aim of determining the physiological role of NADPH oxidases in germination and pollen tube growth, the NOX inhibitor DPI, which has been shown to inhibit both mammalian and plant oxidases (Frahry and Schopfer, 1998; Chen et al., 2013; Altenhöfer et al., 2015), was added to the germination medium. Such addition led to a dramatic reduction in the staining due to superoxide radical. In order to discriminate between NADPH oxidase and peroxidase activities as O_2_^•−^ sources (Carter et al., 2007), we assayed the sensitivity of the staining to sodium azide, which has been described to inhibit different peroxidases (Ortiz de Montellano et al., 1988; Tuisel et al., 1991; Gabison et al., 2014). In this case, no inhibition of the staining was detected (**Figure 6E**). Likewise, germination ratio was 4-fold reduced when the samples were treated with DPI at the very beginning of the *in vitro* germination process (**Figure 7**). NADPH oxidase activity resulted equally critical for pollen tube growth as tube length effectively decreased when the inhibitor was added during the *in vitro* germination process (**Figure 7**). In agreement with the results described above, NADPH oxidase enzymatic activity observed after native PAGE (**Figures 8A, C**) experienced some decrease upon pollen hydration and then was progressively enhanced throughout olive pollen *in vitro* germination when compared to the mature pollen grain. The DPI addition inhibited this activity, while the in-gel activity was not affected by azide (**Figures 8B, D**).

**Figure 9:**
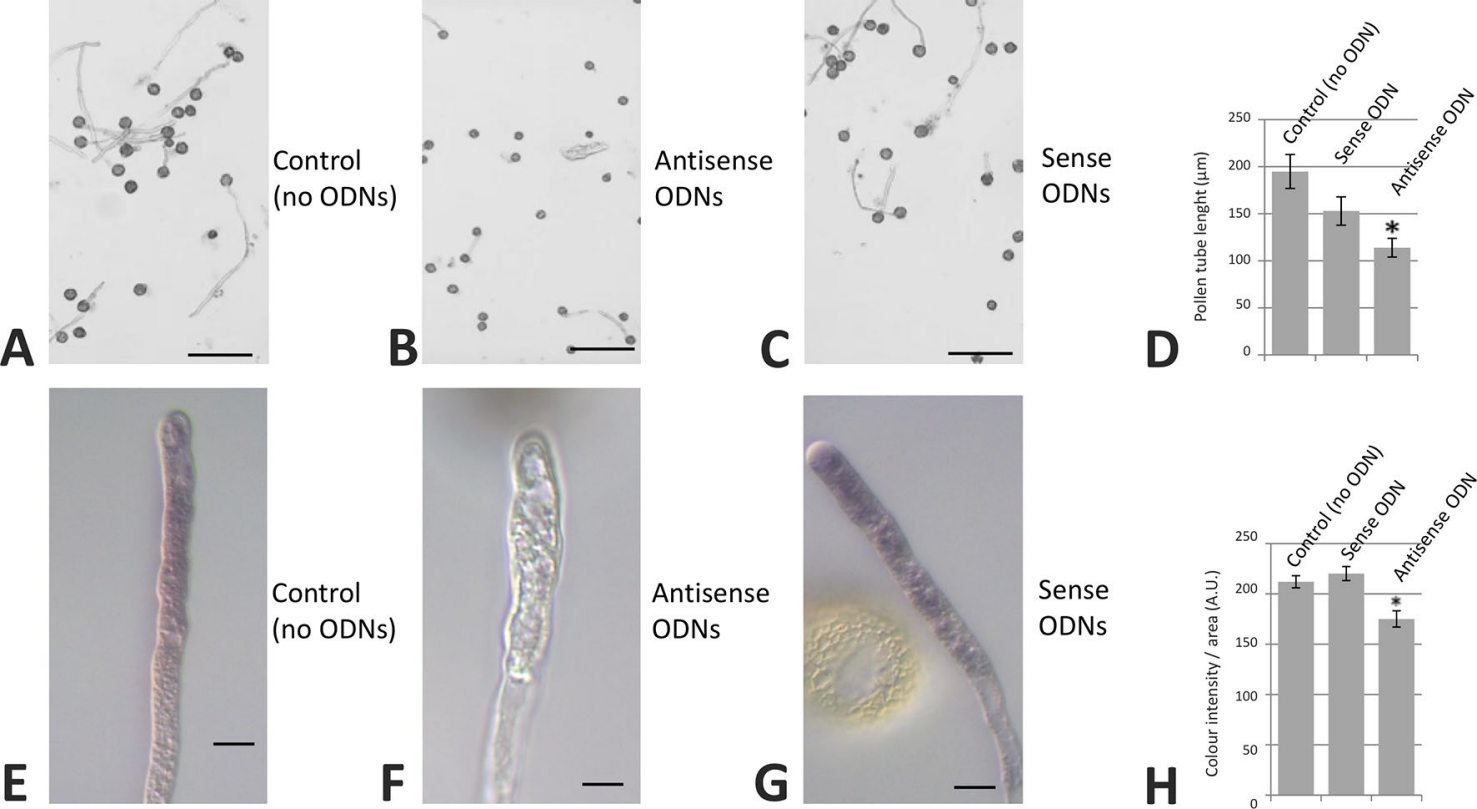
Transfection of olive pollen tubes with OeRbohH-specific antisense oligodeoxynucleotides (ODNs). The OeRbohH sequence obtained was used to design antisense ODNs. Control samples **(A, E)**. Transfection of olive pollen tubes with such antisense ODNs resulted in pollen tube growth inhibition **(B)** as well as in a reduction of the production of tip-localized O_2_^•−^ **(F)** when compared with the control samples or the sense ODNs **(C, G)**. Quantification of pollen tube lengths **(D)** and precipitate intensity **(H)**. Data represent means ± SEM; n = 300. *indicates that the mean value is significantly different from the control at P < 0.05. Bars in upper panel = 100 µm. Bars in bottom panel = 10 µm.

We also carried out olive pollen transfections with specific sense/antisense ODNs corresponding to the OeRbohH ORF sequence. Although a slight difference in pollen tube length average was observed when germinated pollen grains were treated with sense ODNs, this difference was not statistically significant. Alternatively, the treatment with antisense ODNs produced a relevant and statistically significant reduction in both the pollen tube growth and the superoxide production, which visualized as a decrease in NBT staining (**Figure 9**).

## Discussion

In this work, we have carried out a complex characterization of a superoxide-producing Rboh-homologous protein from olive pollen (OeRbohH), which allowed us to identify it as a key protein involved in both pollen germination and pollen tube growth.

Studies focused on ROS production in pollen from different species have revealed that the enzymatic origin of these chemicals is controversial, as well as the localization of these oxygen metabolism products. In fact, it has been suggested that the classically used NOX-inhibitor DPI may cause tube growth inhibition not just because it could be affecting NADPH oxidase activity but also inhibition of other flavoenzymes (Lassig et al., 2014). According to this view, the activity of mitochondrial NAD(P)H dehydrogenases has been suggested to be involved in ROS production in lily (*L. formosanum*) pollen (Cardenas et al., 2006). The presence of different sources of ROS may also suggest different localizations of the product. In lily, ROS are detected in the subapical region of the pollen tube, a cell position also coincidental with most of mitochondria present in this structure. Contrary to that, in tobacco growing pollen tubes, ROS are concentrated at the pollen tube tip (Potocky et al., 2007), and NADPH oxidase has been proposed as the source for superoxide. Moreover, in *Arabidopsis* pollen, and although NADPH oxidase-produced ROS were detected at the tip, the signal was higher in the shank of the pollen tube (Boisson-Dernier et al., 2013), and the authors suggested mitochondria as well as peroxisomes as the possible origins for these chemical species. This dual pollen-ROS source was also suggested in other study carried out in *P. meyeri* (Liu et al., 2009). Furthermore, in kiwifruit pollen tube, there is not a clear tip pattern of ROS generation (Speranza et al., 2012), whereas in cucumber, ROS are detected at the apex during germination onset but in the whole tube throughout the progress of the pollen tube growth (Sirova et al., 2011). Also, a double localization is proposed for pollen specific Rbohs: RbohH and J are both detected in the subapical region, but RbohJ was also located in the pollen shank (Lassig et al., 2014). Thus, further analyses are needed to clarify these potential species-related differences.

We decided to investigate the involvement of Rboh proteins in the sexual plant reproduction of the non-model species *O. europaea* L., not only due to its economic importance but also considering the proposed link between the ROS produced by pollen-intrinsic NADPH oxidase activity and the allergic inflammatory response (Bacsi et al., 2005; Dharajiya et al., 2008). Olive pollen allergy is an especially important disease in the case of olive in Mediterranean countries (Liccardi et al., 1996). This possible pollen NADPH oxidase ability to trigger allergy symptoms (Pazmandi et al., 2012) has already been suggested for other allergenic pollen grains owning NADPH oxidase activity (Boldogh et al., 2005; Wang et al., 2009), although disagreeing studies are also found (Shalaby et al., 2013). OeRbohH must be also considered the first Rboh protein involved in sexual plant reproduction characterized in a tree (Potocky et al., 2007; Kaya et al., 2014).

One of the first challenges to develop this work was the lack of available genomic databases from olive at the time of the onset of the study. This fact forced us to identify conserved domains from different plant Rbohs to design degenerated primers. These included three Rboh sequences, which were decisive for such propose. We initially isolated a first coding fragment, and then, using a variety of PCR-based methodologies, we were able to obtain both the coding and the whole genomic sequences of a Rboh-homologous protein from olive pollen. According to sequence similarity and intron positions, our translated protein can be clustered in the subgroup of plant Rboh proteins showing preferential expression in pollen grain, where AtRbohJ and AtRbohH from *A. thaliana* and a NtNOX from tobacco are included (Potocky et al., 2007; Boisson-Dernier et al., 2013). According to this similarity, the olive sequence was also named as OeRbohH, due to the high level of identity with pollen AtRbohH from *A. thaliana*. These results will be reviewed after the recent release of an olive genome draft (Cruz et al., 2016), which allowed retrieving 12 genomic sequences similar to Rboh (unpublished results), together with the implementation of an improved olive reproductive transcriptome, now underway after the generation of new Illumina sequencing reactions and RNA-Seq analysis. Such new data will allow assessing the possibility of other OeRboh genes being expressed in the anther tissues at prior stages of pollen development or even at the mature pollen grain itself, as the result of the higher reliability and the increased number of readings.

Using a PCR-walking approach, we also obtained a fragment of 1.8kb from the 5-upstream region of OeRbohH. However, this regulatory fragment was not able to yield detectable GUS activity in the generated transgenic lines of *A. thaliana*. Although this result was initially intriguing, we thought it was due to a low gene level expression of OeRbohH. This suggestion agrees with the low-level expression that we found during the PCR quantification experiments. According to this fact, it must be mentioned that a pollen-specific Rboh promoter analysis was included in a study focused on low expressed genes in *Arabidopsis* (Xiao et al., 2010). This low expression was also revealed for OsRbohH in a previous study (Wong et al., 2007). In addition, several Rboh genes from *Medicago truncatula* were not included in a promoter study by GUS fusion approach, because of their very low gene expression levels (Marino et al., 2011). We have attempted different approaches focused to the detection of low GUS activity in order to improve histochemical detection (Rech et al., 2003), with identical negative results.

OeRbohH was almost exclusively expressed in pollen and anthers. Similarly, Kaya et al. (2014) showed that AtRbohH as well as AtRbohJ are specifically expressed in the pollen grain and pollen tubes of *Arabidopsis* flowers. Curiously, we also noticed a very low level of expression in seeds and seedling roots, which were only observed when a high number of PCR cycles were used (not shown).

We have also shown here the occurrence of OeRbohH during olive pollen ontogeny. The results are in accordance with a previous study where superoxide production was detected in the rice anther in a stage-dependent way, with the localization restricted to tapetal cells and microspores (Hu et al., 2011). These authors detected a low level of superoxide anion before meiosis, followed by a noticeable increase during the formation of the tetrads (anther developmental stage 8), and especially when the free microspores were released (stage 9). The following stages showed again a low amount of superoxide, until the rise of mature pollen (stage 12), exhibiting increased superoxide content once again. This pattern is in line with our results regarding superoxide localization (Zafra et al., 2010) in the olive anthers through flower development and indirectly supports our findings concerning OeRbohH gene expression and immunohistochemistry.

OeRbohH immunolocalization during pollen ontogeny is in good agreement with the results obtained by PCR. However, we cannot exclude that the signal may also correspond to cross-reaction with other Rboh proteins present in gametophytic and/or sporophytic germlines. In fact, the importance of the Rboh homologous *RbohE* in the tapetal programmed cell death to proper pollen development has been recently highlighted (Xie et al., 2014).

In addition, different transcriptomic data also indicated that AtRbohH and AtRbohJ increased their expression levels throughout the microgametogenesis, mainly after the second pollen mitosis originating tricellular pollen (Honys and Twell, 2004). According to our results, the present work would be the first evidence about the presence of pollen RbohH proteins during pollen ontogeny in both the gametophytic and sporophytic tissues of the anther.

The presence of transmembrane domains as well as the protein size initially represented a challenge for the recombinant expression of OeRbohH and the subsequent purification process. A recombinant Rboh-type protein from *Arabidopsis* was successfully obtained by Zhang et al. (2009), and we were encouraged to carry out a similar approach. However, the obtained results were different, as RbohH was *in vivo* digested by the host (*E. coli*) during the recombinant expression, yielding three protein fragments mainly identified in the non-soluble protein fraction. This fact was used (after band identifications) to obtain a polyclonal antibody, which ultimately helped us to obtain several important results and conclusions.

Using the polyclonal antibody anti-OeRbohH as well as the YFP fusions, we have shown the occurrence of RbohH in plasma membrane, as it has previously been described for other Rboh isoforms (Keller et al., 1998; Sagi and Fluhr, 2001; Wong et al., 2007; Takeda et al., 2008) and for AtRbohH and J (Boisson-Dernier et al., 2013; Kaya et al., 2014). YFP fluorescence was detected at the plasma membrane and also intracellularly in the endomembrane system with analogous pattern for both constructs, suggesting that the position of the tag does not affect fusion protein localization. Together with the plasma membrane localization, the isoform RbohF is associated with internal membranes (Drerup et al., 2013). Intracellular presence of NtRbohD was also reported in the form of what the authors (Noirot et al., 2014) called rings (identified as Golgi) and dots (suggested to be exocytic compartment). In addition, Lassig et al. (2014) localized *Arabidopsis* pollen Rbohs also at the cytoplasm. Previous Western blotting assays of Rbohs after membrane fractionation drove to a weak signal in internal membrane fractions, which was considered a contamination by the authors (Heyno et al., 2008). In mammals, NOX2 was found at the plasma membrane as well as in endosomes, and NOX4 was described to accumulate in intracellular membranes, the endoplasmic reticulum, and the nuclear compartment (Ushio-Fukai, 2006). However, the presence of NADPH oxidases associated with endomembrane system is not as well documented in plant as in animals.

Our data indicate a critical role of OeRbohH in the control of pollen germination and pollen tube elongation. Initially, we detected a decrease of NADPH oxidase activity when olive mature pollen became hydrated and this activity then raised during the whole process of germination. This activity has been suggested to be involved in changes in the mechanical features of the pollen cell wall during pollen elongation (Smirnova et al., 2014). Contrary to the NADPH oxidase activity, no significant differences were found in OeRbohH gene expression with a relative rise after 1 h of *in vitro* germination. These steady gene expression levels are in good agreement with the data extracted from transcriptome analysis previously achieved in *Arabidopsis* pollen *in vivo* and through semi-*in vivo* germination, and pollen tube growth (Wang et al., 2008; Qin et al., 2009).

The presence and activity of regulatory molecules and networks able to modulate OeRbohH activity are likely to be behind the differences between enzyme RNA expression and enzyme activity. As mentioned before, the production of superoxide by OeRbohH and other RbohHs has been demonstrated to be modulated by multiple factors including Ca^2+^ signaling, acidic signaling phospholipids, and small GTP-binding proteins from the Rac/Rop family (Potocky et al., 2012). Moreover, we have evidence of OeRbohH regulation by *S*-nitrosylation events, which may exert changes in its function (Jimenez-Quesada et al., 2017a; Jimenez-Quesada et al., 2017b). Such PTM is highly dependent on the levels of NO (GSNO) (Corpas et al., 2013), which are critically acting on olive pollen germination physiology (Jimenez-Quesada et al., 2017a; Jimenez-Quesada et al., 2017b).

We established a link between RbohH-produced superoxide and pollen tube growth using specific antisense ODNs and DPI, indicating a key role of RbohH in pollen physiology. NADPH oxidase inhibitor DPI was able to prevent olive pollen germination and superoxide production. This is in agreement with previous results described in tobacco (Potocky et al., 2007), although it does not take place in cucumber, where the NADPH oxidase inhibitor was, on the contrary, able to promote the germination (Sirova et al., 2011). During olive pollen tube growth, we detected superoxide at the tip, with a minor fraction of tubes lacking the NBT stain (about 20% of them), as it has been previously noted also in tobacco pollen tubes (Potocky et al., 2007). The occurrence of NBT staining close to the callose plugs, and in their proximal side only (as referred to the pollen grain), which is described here for the first time, occurred in a significant proportion of such structures. Different explanations for this localization can be proposed. First of all, NBT staining might be the result of superoxide accumulation rather than indicative of superoxide production, as the result of the physical impediment of superoxide transit caused by the callose plugs. Callose synthases have been localized in close proximity to these structures, taking part in their synthesis (Cai et al., 2011). Also, it has been suggested that the activity of callose synthases is enhanced by GTPases like RabA4c, at least during pathogen-induced callose biosynthesis (Ellinger et al., 2014). Because GTPases (at least small GTPases) have been also proposed to induce positively the activity of Rbohs (Potocky et al., 2012), an accumulation of superoxide concomitant to callose synthesis might be foreseen. However, these hypotheses have to be further analyzed experimentally. Our results may also indicate that both the presence of the NADPH oxidase enzyme and likely its activity and consequent superoxide accumulation are polarized, as they occur in the proximal side of the callose plugs in reference to the pollen grain (and in parallel, at the pollen tube tip), likely addressing pollen tube metabolism and direction of growth.

Antisense ODN knockdown strategy inhibited both superoxide production and olive pollen tube growth, as it occurred with the NADPH oxidase inhibitor, indicating a key role of RbohH in such processes. These observations support the hypothesis of OeRbohH as the main responsible of the NADPH oxidase activities and signals found in all previous experiments, and the most important source of O_2_^•−^ during olive pollen germination and tube growth. However, we cannot reject the hypothesis of other flavoenzymes also prone to inhibition by DPI, which might be involved in pollen tube growth. This idea is consistent with the proposal of mitochondria or peroxisomes as ROS sources in other pollen species (Cardenas et al., 2006; Boisson-Dernier et al., 2013).

The idea of RbohH as a mere pollen tube growth promoter is incomplete according to our opinion, considering that the over-expression analysis was able to produce plasma membrane invaginations and abnormal pollen tubes and, finally, tube growth prevention. Furthermore, onion cells expressing the GFP-RbohH fusion were severely disturbed, probably due to the toxic effect of the protein (data not shown). Alterations of pollen tube tip integrity by pollen-Rboh over-expression have been recently reported and connected to over accumulation of cell-wall material (Boisson-Dernier et al., 2013). Altogether, our data are consistent with the idea of RbohH as source of ROS in olive growing pollen tube, and as a key element in controlling tube expansion, interacting with a plethora or other numerous factors, including cGMP, Ca^2+^, ions, and the multitasked signaling gas nitric oxide (Domingos et al., 2015).

## Data Availability

The datasets generated for this study can be found in GenBank and ReprOlive database (http://reprolive.eez.csic.es/olivodb/), GenBank KX648357.

## Author Contributions

JA, VŽ, and MJ-Q designed the work. JA, MJ-Q, JT, and MP performed most of the experimental contributions and data acquisition. JA, VŽ, and MP implemented interpretation of the results and critical review and reprogramming of experiments. All authors contributed to drafting the work, revised the final manuscript and approved submission.

## Funding

This study was supported by the following European Regional Development Fund (ERDF) co-funded grants: BFU2011-22779, BFU2016-77243-P, CSIC 201840E055 and RTC-2017-6654-2, the Ministry of Education, Youth and Sports of CR from ERDF-Project "Centre for Experimental Plant Biology": No. CZ.02.1.01/0.0/0.0/16_019/0000738, Czech Science Foundation GACR 18-18290J, and by a bilateral research agreement between the Spanish National Research Council (CSIC) and the Academy of Sciences of the Czech Republic (AVCR), Ref. 2010CZ0001.

## Conflict of Interest Statement

The authors declare that the research was conducted in the absence of any commercial or financial relationships that could be construed as a potential conflict of interest.
