# Supplementary material for: Generation of Superoxide by OeRbohH, a NADPH Oxidase Activity During Olive (Olea europaea L.) Pollen Development and Germination
Source: Front Plant Sci. 2019 Sep 19;10:1149. doi: 10.3389/fpls.2019.01149 (PMC6761571; doi:10.3389/fpls.2019.01149)
Supplement: Supplementary file 1 [file DataSheet_1.pdf]

Supplementary Material

Generation of superoxide by OeRbohH, a NADPH oxidase activity during olive (Olea europaea L.) pollen development and germination.

María José Jimenez-Quesada, José Angel Traverso, Martin Potocký, Viktor Žárský and Juan de Dios Alché*

*** Correspondence:** Juan de Dios Alché juandedios.alche@eez.csic.es

```
                        OeRboh1804F
                      ------------------------->
AtrbohH  1787 AAAATTTGAATGGCATCCATTCTCCATCACTTCTGCACCAGGAGATGACTATTT 1841
AtrbohJ  1817 CAAATTCGAATGGCATCCATTCTCCATCACTTCTGCACCCGGAGATGAATATTT 1871
PtB9HCK1 1823 AAGTTTTGAATGGCATCCCTTCTCCATCACTTCTGCACCAGGAGATAACAATTT 1877
PtB9IG58 1730 CAAGTTTGAATGGCATCCCTTCTCCATCACTTCTGCACCAGGAGATAACAATTT 1784
OsA2WWR0 1709 TCCTTTCGAATGGCATCCCTTCTCCATCACTTCTGCACCTGGAGATGACTACCT 1763
OsQ8S1T0 1709 TCCTTTCGAATGGCATCCCTTCTCCATCACTTCTGCACCTGGAGATGACTACCT 1763
                  ** *********** ********************* ****** *  *  *

AtrbohH  2249 TCAAAGAGCATATTTTTTTTGGGTGACAAGAGAACAAGCTTCCTTTGATTGGTT 2303
AtrbohJ  2327 ACAAAGAGCATATTTTTATTGGGTGACAAGAGAACAAGCTTCTTTCGAATGGTT 2381
PtB9HCK1 2129 AGAAAGAGCATACTTTTACTGGGTCACAAGAGAACAAAGCTCTTTTGATTGGTT 2183
PtB9IG58 2147 AGAAAGAGCATACTTTTACTGGGTCACAAGAGAACAAAGTTCTTTTGATTGGTT 2201
OsA2WWR0 2120 AGGAAGAGCTTACTTCTACTGGGTGACCAGAGAGCAAGGGTCCTTCGAGTGGTT 2174
OsQ8S1T0 2120 AGGAAGAGCTTACTTCTACTGGGTGACCAGAGAGCAAGGGTCCTTCGAGTGGTT 2174
                 ****** ** ** *  ***** ** ***** ***    ** ** ** *****
                    <-----------------------------
                            OeRboh2286R
```

Supplementary Figure 1. Partial ORF alignment of homologous plant NOXs *from A. thaliana* (At5g60010, At3g45810), *P. trichocarpa* (PtB9HCK1, PtB9IGS8) and *O. sativa* (OsA2WWR0, OsQ8S1T0). Arrows indicate the conserved domains used to design degenerated primers forward and reverse.

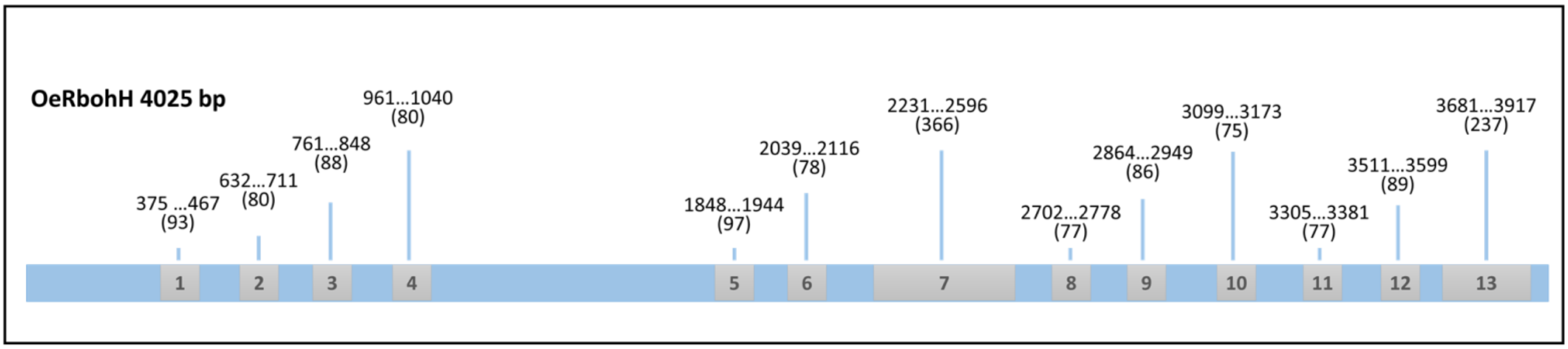

Supplementary Figure 2. Genomic sequence of olive pollen *OeRbohH* with introns location (grey boxes). Location and length in bp (in brackets) are indicated.

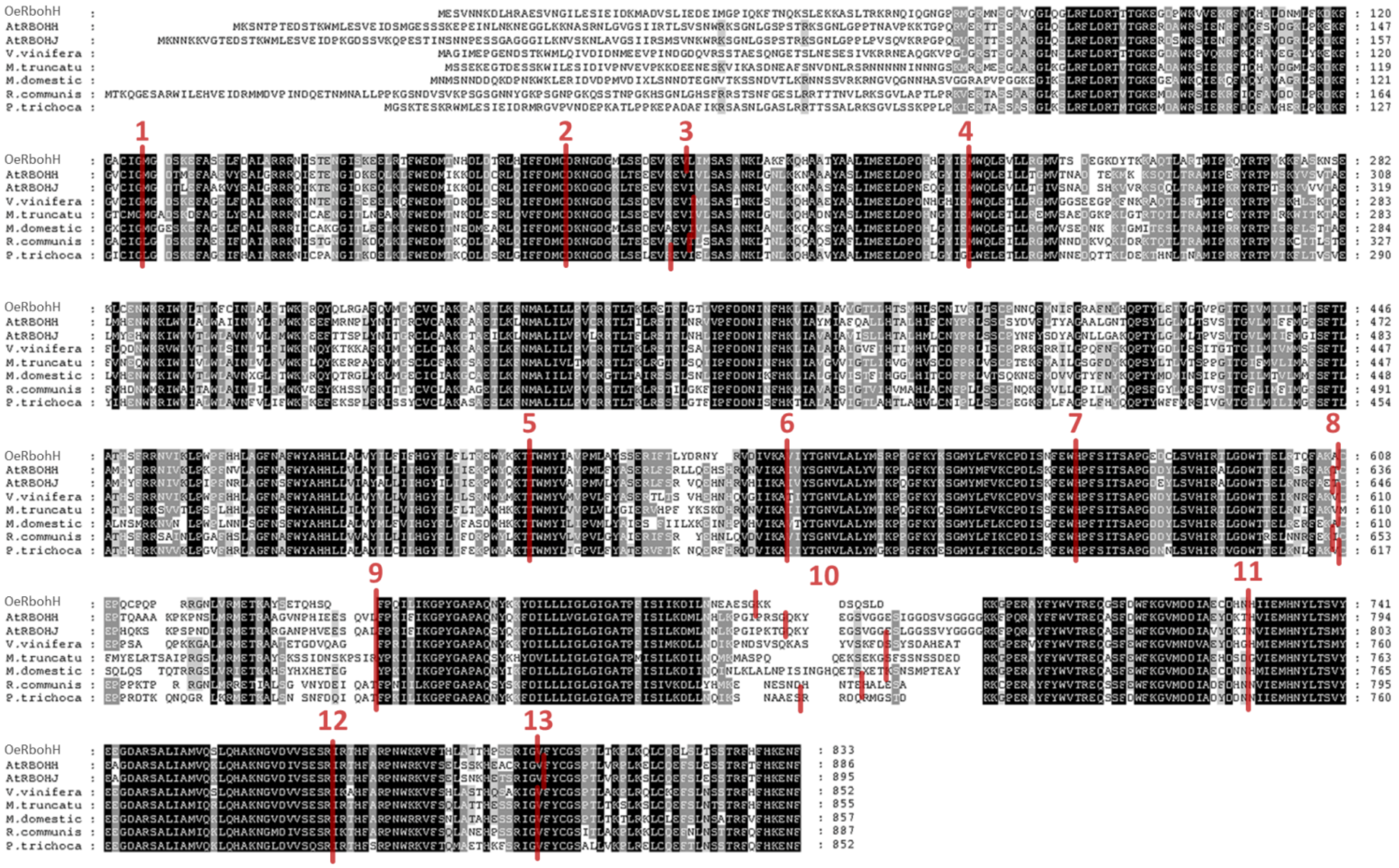

Supplementary Figure 3. OeRbohH introns are conserved. Amino acid alignment of Rbohs from *A. thaliana*, *V. vinifera*, *M. truncatula*, *M. domestica*, *R. communis*, and *P. trichocarpa*. Introns location is conserved (red boxes).

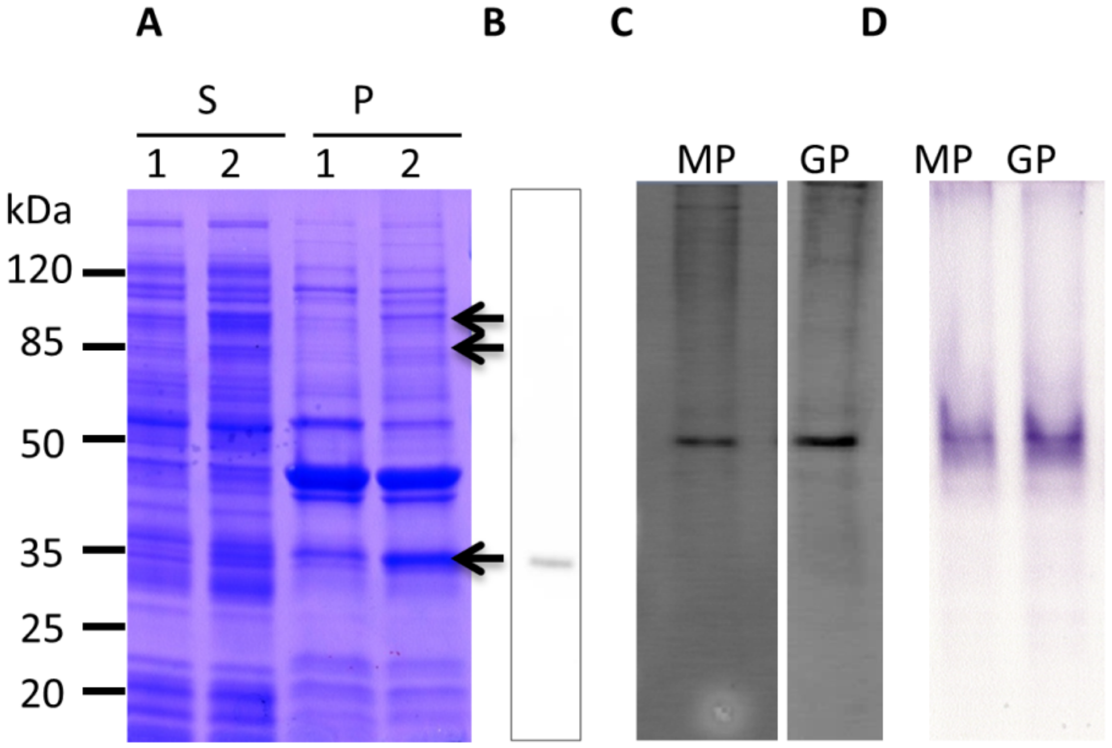

Supplementary Figure 4. (A) SDS-PAGE of crude protein extracts of *E.coli* after induction of the expression. S: soluble fraction. P: precipitate; 1: negative control (pET51b); 2: expression construct (pET51b-OeRbohH). Arrows indicate the 3 bands which were later identified by LC-ESI-MS as such OeRbohH. (B) Western-blot using an anti-HisTag antibody in the insoluble fraction of the extract. (C) Western–blot using the anti–OeRbohH antibody in olive pollen protein extracts subjected to native-PAGE. The imuno-reactive band in the blot match the NADPH oxidase activity band in parallel in gel activity assays (D). MP, mature pollen; GP: germinated pollen.

Primer name	Sequence	Use
OeRboh1804F	GAATGGCATCCMTTCTCCATCACTTC	Degenerated primer plant Rbohs
OeRboh2286R	TCTCTKGTSACCCARWARAARTAWGCTCTT	
UPM long	CTAATACGACTCACTATAGGGCAAGCAGTGGTATCAACGCAGAGT	Universal Primer for RACE 5' y RACE 3'
UPM short	CTAATACGACTCACTATAGGGC	
PoliT24(A)	TTTTTTTTTTTTTTTTTTTTTTTTN	Race 3'
OeRbohFW2	CTAATTGGTTTGGGAATTGGAGCAACCCC	RACE 3'
OeRbohRW2	CTGACTAAGTTTCCCCTTCTTGGTTGTGGACACTGAGG	RACE 5'
OeXbaRbohF	CGCTCTAGAATGGAATCAGTGAACAATAAAGATC	For coding genomic, pET51b and GFP vector cloning
OeSacRbohR	GCCGAGCTCCAAAGTTTTCCTTGTGAAAATG	For coding genomic and pET51b vector cloning
UTRRbohF	ATTGATATTCGTACTAGTATCTCAACAG	Gene expression studies
UTRRbohR	AAAATGCGAGAATTTTACAAGTATTCAC	
Oe18S-F	TTTGATGGTACCTGCTACTCGGATAACC	Olive Housekeeping gene (ribosomal 18S)
Oe18S-R	CTCTCCGGAATCGAACCCTAATTCTCC	
OeA-F	TTGCTCTCGACTATGAACAGG	Olive Housekeeping gene (actin)
OeA-R	CTCTCGGCCCCAATAGTAATA	
OeqF	AGTGTCCACAACCAAGAAGG	For quantitative PCR
OeqR	ATGGGGTTGCTCCAATTC	
OeRbohR	ACGTTCTAGAGAAAGTTTTCCTTGTGAAAATG	For GFP Vector cloning
OeRboh-Ngo-F	ATAGCCGGCATGGAATCAGTGAACAAT	For YFP Vector cloning
OeRboh-Xma-RS	TATCCCGGGTCAAGCTCCAAAGTTTTC	
OeRboh-Xma-RNS	TATCCCGGGTCCAGCTCCAAAGTTTTC	
OeRbohRW8	CCCATTTACAGATTCTGCTCTATGTAGATCTTTATTGTTC	For OeRbohH promoter isolation
OeRbohRW7	GAAACATCAGCCATTTTATCAATCTCAATGCTCTCC	
OeRbohRW9	ATGAATTGTCCTGTCAAGGTGCTTATTGAGTCTTAGC	
OeRbohRW10	CGTCTCTCAAGATACAGCGAAATCTCCTTGCAGCAG	
pRboh1.8F	ATACTCGAGGCCCGGGGAGGTAT	For pBI101 cloning
pRboh1.5F	TGCACTCGAGCGGGGAGGTACTTCTGCT	
pRboh0.7F	GCGCTCGAGTCCGAATTACGAGT	
pRboh0.2F	TGCACTCGAGGTAAGTTGGGGATCT	
pRbohR	TGCAAGATCTGACACTGACTGATTTCTCTGTTGAG	

Supplementary Table 1. List of primers used in this study.

	PROTEIN HITS	NAME [ORGANISM]	PEPTIDE
1	gi|56201942	Putative respiratory burst oxidase [*Oryza sativa Japonica* Group]	R.IGVFYCGSPTLTK.Q
			R.SALIAMVQSLQHAK.N
2	gi|225435144	PREDICTED: putative respiratory burst oxidase homolog protein H-like [*Vitis vinifera*]	K.NGVDVVSESR.I
			K.FNMALILIPVCR.R + Oxidation (M)
			R.SALIAMVQSLQHAK.N
			R.SALIAMVQSLQHAK.N + Oxidation (M)
	gi|171903618	Respiratory burst oxidase-like protein J [*Hordeum vulgare subsp. vulgare*]	R.IGVFYCGSPTLTK.T
			R.SALIAMVQSLQHAK.N
			R.SALIAMVQSLQHAK.N + Oxidation (M)
3	gi|109631188	NOX1 [*Striga asiatica*]	K.NGVDVVSESR.I
			R.EQGSFDWFK.G
			R.IGVFYCGSPTLTKPLK.K
	gi|225435144	PREDICTED: putative respiratory burst oxidase homolog protein H-like [*Vitis vinifera*]	R.FHFHK.E
			K.NGVDVVSESR.I
			R.SALIAMVQSLQHAK.N
	gi|357131247	PREDICTED: putative respiratory burst oxidase homolog protein H-like [*Brachypodium distachyon*]	R.FHFHK.E
			R.EQGSFDWFK.G
			R.SALIAMVQSLQHAK.N
	gi|357505521	Respiratory burst oxidase-like protein [*Medicago truncatula*]	R.FHFHK.E
			K.NGVDVVSESR.I
			R.THFARPNWK.K
	gi|294462658	Unknown [*Picea sitchensis*]	R.EEGSFDWFK.G
			R.SALIAMLQALNHAK.H + Oxidation (M)
			K.GIIELHNYCTSVYEEGDAR.S
	gi|18389310	Respiratory burst oxidase protein F [*Solanum tuberosum*]	R.EQGSFDWFK.G
			R.THFARPNWK.K
	gi|166199744	RecName: Full=Respiratory burst oxidase homolog protein D; AltName: Full=NADPH oxidase RBOHD; AltName: Full=StRBOHD	R.EQGSFDWFK.G
			R.SALIAMLQSINHAK.N
	gi|356527324	PREDICTED: respiratory burst oxidase homolog protein C-like isoform 1 [*Glycine max*]	R.EQGSFDWFK.G
			R.SALIAMLQSLNHAK.N
	gi|326491643	Predicted protein [*Hordeum vulgare subsp. vulgare*]	R.SALIAMLQSLNHAK.H
			K.GIIELHNYCTSVYEEGDAR.S
	gi|168011242	Predicted protein [*Physcomitrella patens subsp. patens*]	K.AYFYWMTR.E
			R.THFARPNWK.S

Supplementary Table 2. Identification from samples 1, 2 and 3 and peptide summary obtained by LC-ESI-MS/MS. Taxonomy: Viridiplantae (Green Plants).
